# Integrative metabolomics reveals unique metabolic traits in Guillain-Barré Syndrome and its variants

**DOI:** 10.1038/s41598-018-37572-w

**Published:** 2019-01-31

**Authors:** Soo Jin Park, Jong Kuk Kim, Hyun-Hwi Kim, Byeol-A. Yoon, Dong Yoon Ji, Chang-Wan Lee, Ho Jin Kim, Kyoung Heon Kim, Ha Young Shin, Sung Jean Park, Do Yup Lee

**Affiliations:** 1 The Department of Bio and Fermentation Convergence Technology, BK21 PLUS Program, Kookmin University, Seoul, 02707 Republic of Korea; 2 Department of Neurology, Peripheral Neuropathy Research Center, Dong-A University College of Medicine, Busan, 49315 Republic of Korea; 3 College of Pharmacy and Gachon Institute of Pharmaceutical Sciences, Gachon University, Incheon, 21936 Republic of Korea; 4 The Department of Neurology, Research Institute and Hospital of the National Cancer Center, Goyang, Republic of Korea; 5 The Department of Biotechnology, Graduate School, Korea University, Seoul, Republic of Korea; 6 Department of Neurology, Brain Korea 21 Project for Medical Science, Yonsei University College of Medicine, Seoul, Korea

## Abstract

Guillain–Barré syndrome (GBS) is an acute fatal progressive disease caused by autoimmune mechanism mainly affecting peripheral nervous system. Although the syndrome is clinically sub-classified into several variants, specific biomarker and exact pathomechanism of each subtypes are not well elucidated yet. In current study, integrative metabolomic and lipidomic profiles were acquisitioned from cerebrospinal fluid samples of 86 GBS from three variants and 20 disease controls. And the data were systematically compared to our previous result on inflammatory demyelination disorders of central nervous system (IDDs) and healthy controls. Primary metabolite profiles revealed unique metabolic traits in which 9 and 7 compounds were specifically changed in GBS and IDD, respectively. Next, the biomarker panel with 10 primary metabolites showed a fairly good discrimination power among 3 GBS subtypes, healthy controls, and disease controls (AUCs ranged 0.849–0.999). The robustness of the biomarker panel was vigorously validated by multi-step statistical evaluation. Subsequent lipidomics revealed GBS variant-specific alteration where the significant elevations of lyso-phosphatidylcholines and sphingomyelins were unique to AIDP (acute inflammatory demyelinating polyneuropathy) and AMAN (acute motor axonal neuropathy), respectively. And metabolome-wide multivariate correlation analysis identified potential clinical association between GBS disability scale (Hughes score) and CSF lipids (monoacylglycerols, and sphingomyelins). Finally, Bayesian network analysis of covarianced structures of primary metabolites and lipids proposed metabolic hub and potential biochemical linkage associated with the pathology.

## Introduction

Guillain-Barre syndrome (GBS) is an acute inflammatory peripheral neuropathy. It is rare, but once it develops, a portion of patients is accompanied by a serious clinical course. Thus, optimal treatment following early diagnostic is critical for prognostic determination^1^. GBS typically occurs by an autoimmune response to a predisposing factor such as infection or vaccinations. Over the past decades, several types of GBS have been identified and recent studies have revealed that some of these subtypes were determined by specific anti-ganglioside antibodies^2^. It included acute inflammatory demyelinating polyneuropathy (AIDP), acute motor axonal neuropathy (AMAN), and Miller Fisher syndrome (MFS) according to clinical, electrophysiological, and immunological information^3–5^. Although several biomarkers and pathogenesis have been recently proposed, precise patho-mechanisms still remain unknown^6,7^. Accordingly, disease specific therapies have not been developed yet, other than intravenous immunoglobulin or plasma exchange^8^. Recently, novel predictive models for treatment options and prognosis of GBS have been suggested based on cutting-edge technology such as microarrays and proteomics^9–11^. The high-throughput molecular profiling has proposed potential biomarkers and patho-mechanisms from CSF or blood samples. However, CSF metabolic profiles in GBS and its subtypes have not been investigated.

Clinical studies on the metabolome are widely applied for discovering diagnostic and prognostic markers, and for uncovering underlying patho-mechanism of diseases including autoinflammatory syndromes^12,13^. Accordingly, our study aimed to investigate unique metabolic profiles of three GBS variants (AIDP, AMAN, and MFS) by applying multiplex instrumental analysis on CSF samples. Integrative profiles of primary metabolites were acquired by GC-TOF MS and NMR, and lipidomic readout was obtained by LC-Oribrap MS. In addition, comparative analysis following batch effect removal process was conducted against healthy controls and IDDs (multiple sclerosis and neuromyelitis optica spectrum disorder) obtained from our recent study^12,14^ in order to delineate disorders of peripheral nervous system from central nervous system.

## Results

### Demographic and clinical data of GBS patients and disease controls

**Figure 1:**
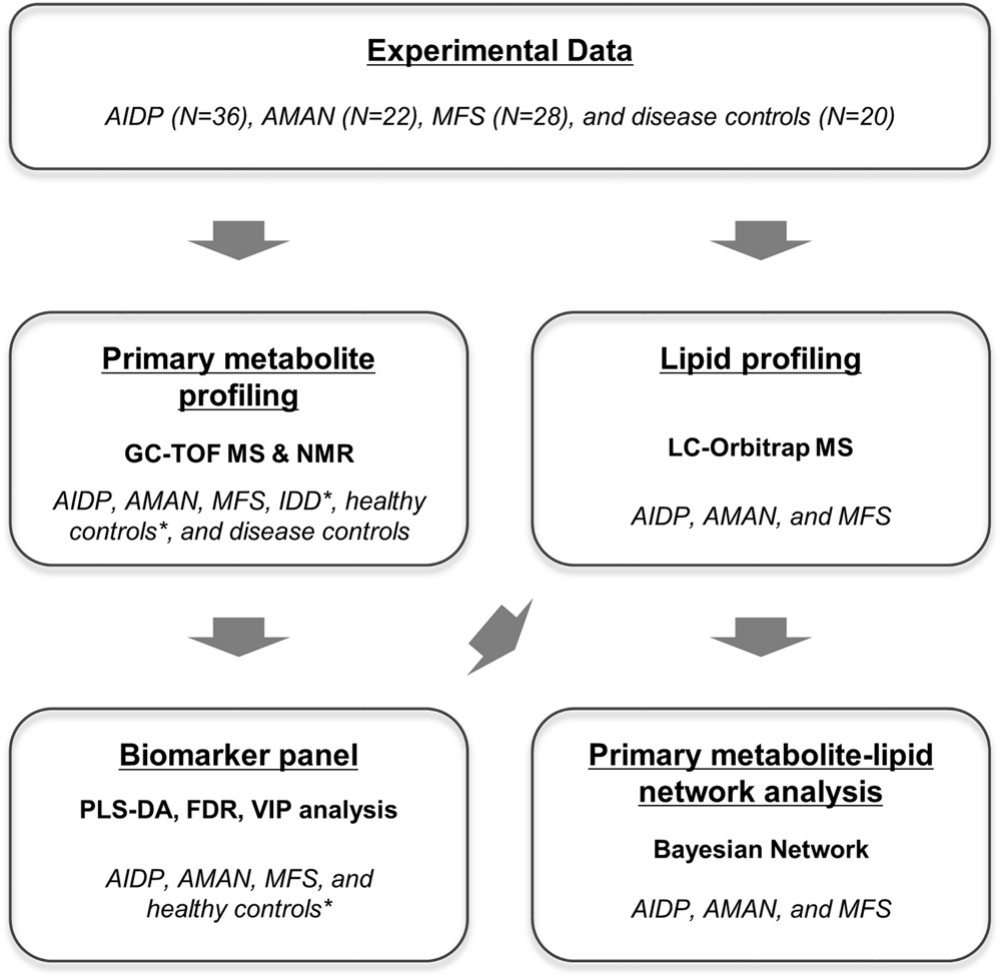
Summary of the sample collection, instrumental analysis (GC-TOF MS, LC-Orbitrap MS, and NMR), data processing, and statistical analysis (including model validation). * indicates the data source from the previous study.

The total study subjects were 106 patients with a mean age of 52.3 ± 17.7 years (male = 54 and female = 52) (Table 1). Case number of AIDP, AMAN, MFS, and disease control were 36 (53.4 ± 18.1, male: female = 21:15), 22 (60.9 ± 11.1, male: female = 7:15), 28 (44.4 ± 16.0, male: female = 15:13) and 20 (52.1 ± 20.0, male: female = 11:9) respectively. Hughes scores were retrospectively collected from the patients with AIDP (n = 25), AMAN (n = 18), and MFS (n = 21). Each final diagnosis of disease control group was idiopathic oculomotor nerve palsy (n = 8), brainstem or spinal cord ischemia (n = 5), idiopathic brachial plexopathy (n = 1), Wernicke encephalopathy (n = 1), Vernet’s syndrome (n = 1), motor neuron disease (n = 1), diabetic polyneuropathy (n = 1), nutrition deficiency syndrome (n = 1), and pineal gland tumor (n = 1). There was no age difference between the groups, but the MFS group was younger than the AMAN group (p = 0.007). Overall workflow was provided in Fig. 1, which included the sample collection, instrumental analysis (GC-TOF MS, LC-Orbitrap MS, and NMR), data processing, statistical analysis, and biomarker model construction.

**Table 1:** Demographic and clinical data of patients with GBS and disease controls.

	AIDP (n, 36)	AMAN (n, 22)	MFS (n, 28)	Other disease (n, 20)	P-value
Age	53.4 (±18.1)	60.9 (±11.1)	44.4 (±16.0)	52.1 ( ± 20.0)	0.013
Sex (Male, %)	58.3%	31.8%	53.6%	55.0%	0.224
Hughes score	2.3 (±1.2)	3.8 (±0.9)	2.3 (±1.0)	N/A	<0.001
Antibody (%)					
IgG anti-GM1	0.0%	100.0%	7.1%	0.0%	
IgG anti-GQ1b	0.0%	0.0%	100.0%	0.0%	

AMAN and MFS patients were selected according to positivity of each representing antibody (IgG anti-GM1 and IgG anti-GQ1b antibody, respectively) for their clear classification and comparison between groups.

AIDP, acute inflammatory demyelinating polyneuropathy; AMAN, acute motor axonal neuropathy; MFS, Miller Fisher syndrome.

### The comparative analysis of CSF metabolic profiles of GBS against healthy controls, disease controls, and IDDs

**Figure 2:**
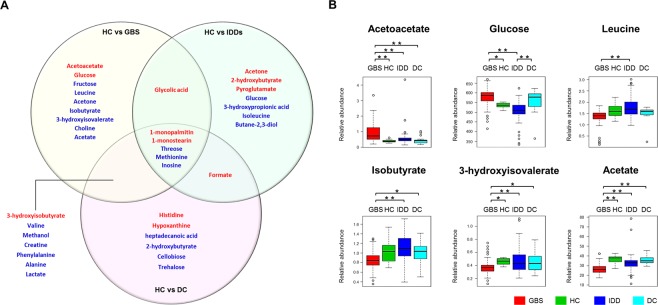
The metabolite list of differential abundances among GBS, IDDs, and disease controls against healthy controls (**A**). Red and blue colored metabolites indicate significant elevation or reduction compared to healthy control (p < 0.05). HC: healthy control; IDDs: inflammatory demyelinating disorder of CNS; GBS: Guillain–Barré syndrome; DC: disease control. Relative abundances of the GBS-specific metabolites (**B**). Boxes show the interquartile range, and whiskers indicate the minimum and maximum of all of the data. Analysis of variance is performed based on *Kruskal-Wallis* test and subsequent *posthoc* analysis. * and ** indicate significance levels, p < 0.05 and <0.01, respectively.

The significance differences were measured in 22 metabolites between GBS patients and healthy controls. The most dramatic difference was observed in acetoacetate that was up to two-times higher in GBS. The next ones were monoacylglycerols (1-monopalmitin and 1-monostearin), 3-hydroxyisobutyrate, and glycolic acid. Among the down-regulated metabolites were methionine, threose, methanol, acetate, and creatine that were present at the highest fold changes. Pathway over-representation analysis showed that glycolysis and amino acid metabolism were the metabolic process under the most significant alteration (Supplementary Fig. S1). The highest centrality was identified in ketone body metabolism by the topological analysis. For identifying the GBS-specific metabolic changes, pair-wise statistical comparison by *Student t-test* was first conducted on GBS, IDDs, and disease controls against healthy controls (Fig. 2). Nine metabolites were attributed as GBS-specificity while 7 and 6 metabolites were categorized as IDD-unique or common between GBS and IDD, respectively. Unique metabolic features of GBS included acetoacetate, glucose, fructose, leucine, acetone, isobutyrate, 3-hydroxyisovalerate, choline, and acetate. *Kruskal-Wallis* test with *Benjamini*-*Hochberg* adjustment demonstrated acetoacetate and acetate were the most characteristic to the GBS group whereas other metabolites partially passed multiple comparison criteria (Fig. 2 and Supplementary Table S1). Acetone, 2-hydroxy butyrate, pyroglutamate, glucose, 3-hydroxypropionic acid, isoleucine, and butane-2,3-diol were specific to IDDs. Among them were acetone and 2-hydroxybutyrate that presented exclusive differences in the IDDs group.

### Unique metabolic dys-regulation of the GBS subtypes

We further investigated the metabolic dys-regulation focusing on uniqueness and similarity among the GBS subtypes (AIDP, AMAN, and MFS). The MFS group presented the widest range of primary metabolic alteration (*t-test*, p-value < 0.05) (Table 2). A total of 43.7% of metabolites were present at different levels in MFS whereas 24.1% and 23.0% of metabolites presented the significant differences in AIDP and AMAN.

**Table 2:** The list of metabolites that present GBS subtype-specific changes (upper panel) and common features.

	AIDP						AMAN						MFS					
	Compound	T-TEST (HC vs AIDP)			Kruskal-Wallis		Compound	T-TEST (HC vs AMAN)			Kruskal-Wallis		Compound	T-TEST (HC vs MFS)			Kruskal-Wallis	
		p-value	FDR	Fold change	p-value	Adjusted p-value (Benjamini/ Hochberg)		p-value	FDR	Fold change	p-value	Adjusted p-value (Benjamini/ Hochberg)		p-value	FDR	Fold change	p-value	Adjusted p-value (Benjamini/ Hochberg)
Unique features													Glycolic acid	3E-03	1E-02	1.9	2E-04	8E-04
													Creatinine	1E-02	4E-02	0.9	1E-02	3E-02
													Ornithine	3E-02	7E-02	0.8	6E-05	4E-04
													Gluconic acid	2E-03	1E-02	0.8	3E-04	1E-03
													Threonine	7E-03	2E-02	0.8	2E-02	4E-02
	Histidine	2E-02	1E-01	1.3	2E-03	5E-03	Tryptophan	2E-02	1E-01	1.4	1E-02	2E-02	Ethanolamine	7E-03	2E-02	0.7	3E-03	6E-03
	Palmitic acid	2E-02	1E-01	1.1	7E-05	4E-04	Lauric acid	4E-03	3E-02	1.4	4E-04	1E-03	Lysine	2E-03	8E-03	0.7	9E-05	4E-04
	Myristic acid	3E-02	1E-01	1.1	1E-03	3E-03	Heptadecanoic acid	3E-02	1E-01	1.2	1E-03	4E-03	Dimethyl sulfone	2E-02	5E-02	0.7	2E-03	5E-03
													Isoleucine	4E-04	3E-03	0.6	2E-04	8E-04
													Proline	6E-04	4E-03	0.5	3E-06	2E-05
													Lyxose	2E-02	6E-02	0.4	2E-02	3E-02
													Pyruvate	0E + 00	0E + 00	0.3	4E-10	4E-08
													Trehalose	4E-02	8E-02	0.1	5E-07	7E-06
													Cellobiose	2E-02	6E-02	0.1	7E-07	8E-06
Common features													Acetoacetate	3E-05	3E-04	2.7	3E-08	8E-07
													1-monostearin	2E-04	2E-03	1.7	3E-08	8E-07
													3-hydroxyisobutyrate	6E-06	8E-05	1.5	8E-05	4E-04
	1-monostearin	2E-05	3E-04	2.0	3E-08	8E-07	1-monostearin	2E-06	9E-05	2.7	3E-08	8E-07	Galactonic acid	2E-02	6E-02	1.4	4E-03	9E-03
	1-monopalmitin	2E-03	2E-02	1.6	5E-05	3E-04	Acetoacetate	2E-04	3E-03	2.6	3E-08	8E-07	1-monopalmitin	1E-03	6E-03	1.4	5E-05	3E-04
	3-hydroxyisobutyrate	5E-03	5E-02	1.4	8E-05	4E-04	1-monopalmitin	2E-05	4E-04	1.9	5E-05	3E-04	Formate	1E-02	3E-02	1.3	3E-07	5E-06
	Stearic acid	1E-02	8E-02	1.1	3E-04	1E-03	3-hydroxyisobutyrate	5E-05	1E-03	1.6	8E-05	4E-04	Glucose	1E-04	1E-03	1.1	1E-06	1E-05
	Glucose	5E-05	8E-04	1.1	1E-06	1E-05	Formate	4E-04	4E-03	1.6	3E-07	5E-06	Myo-inositol	4E-03	1E-02	0.9	2E-02	4E-02
	Isobutyrate	2E-02	1E-01	0.9	8E-02	1E-01	Galactonic acid	1E-02	8E-02	1.4	4E-03	9E-03	Glutamine	1E-02	3E-02	0.9	1E-03	3E-03
	Fructose	4E-02	2E-01	0.8	2E-02	4E-02	Stearic acid	4E-03	3E-02	1.2	3E-z04	1E-03	Valine	4E-02	8E-02	0.8	1E-03	3E-03
	Leucine	8E-03	7E-02	0.8	2E-02	3E-02	Glutamine	1E-02	7E-02	0.9	1E-03	3E-03	Choline	2E-02	6E-02	0.8	1E-01	2E-01
	Lactate	2E-06	6E-05	0.8	2E-08	8E-07	Myo-inositol	2E-02	1E-01	0.9	2E-02	4E-02	Lactate	6E-12	3E-10	0.8	2E-08	8E-07
	3-hydroxyisovalerate	6E-03	6E-02	0.8	1E-03	3E-03	Phenylalanine	4E-02	2E-01	0.8	8E-04	3E-03	Isobutyrate	5E-03	2E-02	0.8	8E-02	1E-01
	Creatine	6E-04	9E-03	0.8	2E-06	2E-05	Alanine	3E-02	1E-01	0.8	2E-05	1E-04	Leucine	9E-03	3E-02	0.8	2E-02	3E-02
	Inosine	1E-02	7E-02	0.8	2E-02	3E-02	Isobutyrate	2E-02	9E-02	0.8	8E-02	1E-01	Fructose	3E-03	1E-02	0.8	2E-02	4E-02
	Choline	4E-02	2E-01	0.8	1E-01	2E-01	Acetate	1E-05	4E-04	0.8	7E-08	1E-06	3-hydroxyisovalerate	1E-03	7E-03	0.8	1E-03	3E-03
	Acetate	2E-06	9E-05	0.8	7E-08	1E-06	Creatine	2E-06	2E-04	0.7	2E-06	2E-05	Inosine	3E-04	2E-03	0.7	2E-02	3E-02
	Phenylalanine	9E-03	7E-02	0.7	8E-04	3E-03	Valine	1E-04	2E-03	0.7	1E-03	3E-03	Phenylalanine	3E-05	3E-04	0.7	8E-04	3E-03
	Methanol	2E-02	1E-01	0.7	5E-03	1E-02	Methanol	3E-04	3E-03	0.6	5E-03	1E-02	Creatine	2E-11	6E-10	0.7	2E-06	2E-05
	Methionine	2E-02	1E-01	0.6	2E-05	1E-04	Threose	6E-04	5E-03	0.6	8E-05	4E-04	Methanol	3E-03	1E-02	0.7	5E-03	1E-02
	Threose	5E-07	4E-05	0.6	8E-05	4E-04							Alanine	1E-08	2E-07	0.6	2E-05	1E-04
													Acetate	6E-11	1E-09	0.6	7E-08	1E-06
													Threose	7E-06	9E-05	0.6	8E-05	4E-04
													Methionine	1E-03	6E-03	0.4	2E-05	1E-04

Statistical evaluation is first done based on *Student’s t-test* against healthy controls. Common features are the metabolites that are present at different levels (with same direction of fold change) at least two comparisons. Analysis of variance is conducted for additional evaluation by *Kruskal-Wallis* test with Benjamini/Hochberg analysis.

Pathway over-representation analysis showed characteristic metabolic dysfunction according to the GBS subtypes (Supplementary Fig. S2). The MFS group showed the most significant alteration in Val-Leu-Ile metabolism, Arg-Pro metabolism, and central carbon metabolism (e.g. glycolysis and pyruvate metabolism). The AIDP group presented that glycolysis were the most significantly altered among the other subtypes followed by fatty acid biosynthesis. Ala-Asp-Glu metabolism, nitrogen metabolism, ketone body metabolism, and taurine metabolism were specific to the AMAN group whereas glycolysis and pyruvate metabolism were common to the MFS and AIDP groups.

Likewise, the most specific changes were found in MFS where 14 compounds were not shared by AIDP, and AMAN (Table 2). The metabolic dys-regulation of MFS was featured by the concomitant down-regulation in the nitrogenous compounds (creatinine, ornithine, leucine, ethanolamine, lysine, isoleucine, proline). Others were glycolic acid, gluconic acid, dimethyl sulfone, lyxose, pyruvate, trehalose, and cellobiose. The AIDP group showed exclusive feature, myristic acid, palmitic acid, and histidine whereas lauric acid, heptadecanoic acid, and tryptophan were unique to AMAN.

Considering multiple testing correction, Kruskal-Wallis test with Benjamini-Hochberg procedure and Man-Whitney test with Bonferroni correction were performed. Overall, the statistical result corresponded to the primary evaluation by *Student’s t-test*. Among them was acetate that presented the significant differences in 5 comparisons of all possible 6 combinations. Furthermore, potential confounding effects of age and gender were evaluated based on *MANCOVA* following normality and *Kruskal-Wallis* tests. After age correction, 78, 77, and 104 were significantly different in AIDP, AMAN, and MFS groups, respectively. Similarly, 70, 74, and 92 metabolites were present at different abundances following gender correction. The results implied the highest level of the primary metabolic perturbation in the MFS group relative to ones of the AIDP and AMAN groups.

### Biomarker signature discriminative of the GBS subtypes

**Figure 3:**
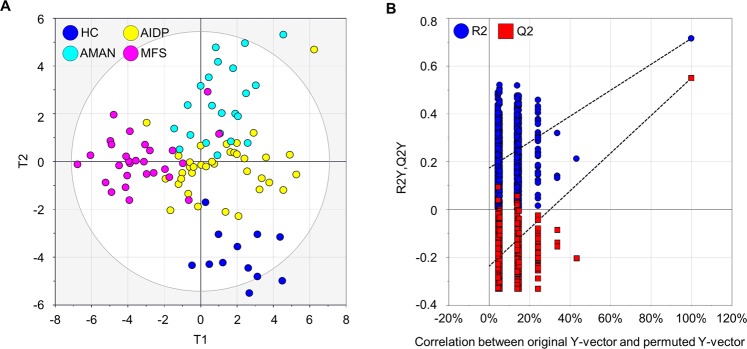
Distinctive metabolic phenotypes among different types of GBS variants and the healthy controls. (**A**) Score scatter plot based on multivariate statistical model by PLS-DA. HC: healthy control. (**B**) Random permutation plot (999 times) on the datasets of the four groups. The vertical axis presents R^2^ (blue points) and Q^2^ (red points) values of the model. The horizontal axis shows the correlation coefficient between the original and the permuted Y-variable.

The metabolites were pre-selected for constructing biomarker recomposite using PLS-DA prior to validation statistics, ROC analysis. The PLS-DA model with seven-fold cross validation demonstrated the high levels of an explained variance (R^2^Y) of 0.628 and predictability (Q^2^Y) of 0.444 (Fig. 3A).

To examine the level of overfitting, which is determined by trade-off between fit (R^2^) vs prediction (Q^2^), random permutation test with 999 iterations was conducted. It provides the statistical significance of the estimated prediction power of the models by comparing R^2^Y and Q^2^Y values between the original model and the randomly re-ordered model^15^. The result showed that Y-axis intercepts of R^2^ and Q^2^ were below 0.3 and 0.05 (Fig. 3B), which met the general criteria of R^2^ <0.3 and Q^2^ <0.05. The validation was also evidenced by the result that all permuted R^2^ and Q^2^ values (the left on x-axis) were lower than the original values on the right^16,17^.

**Figure 4:**
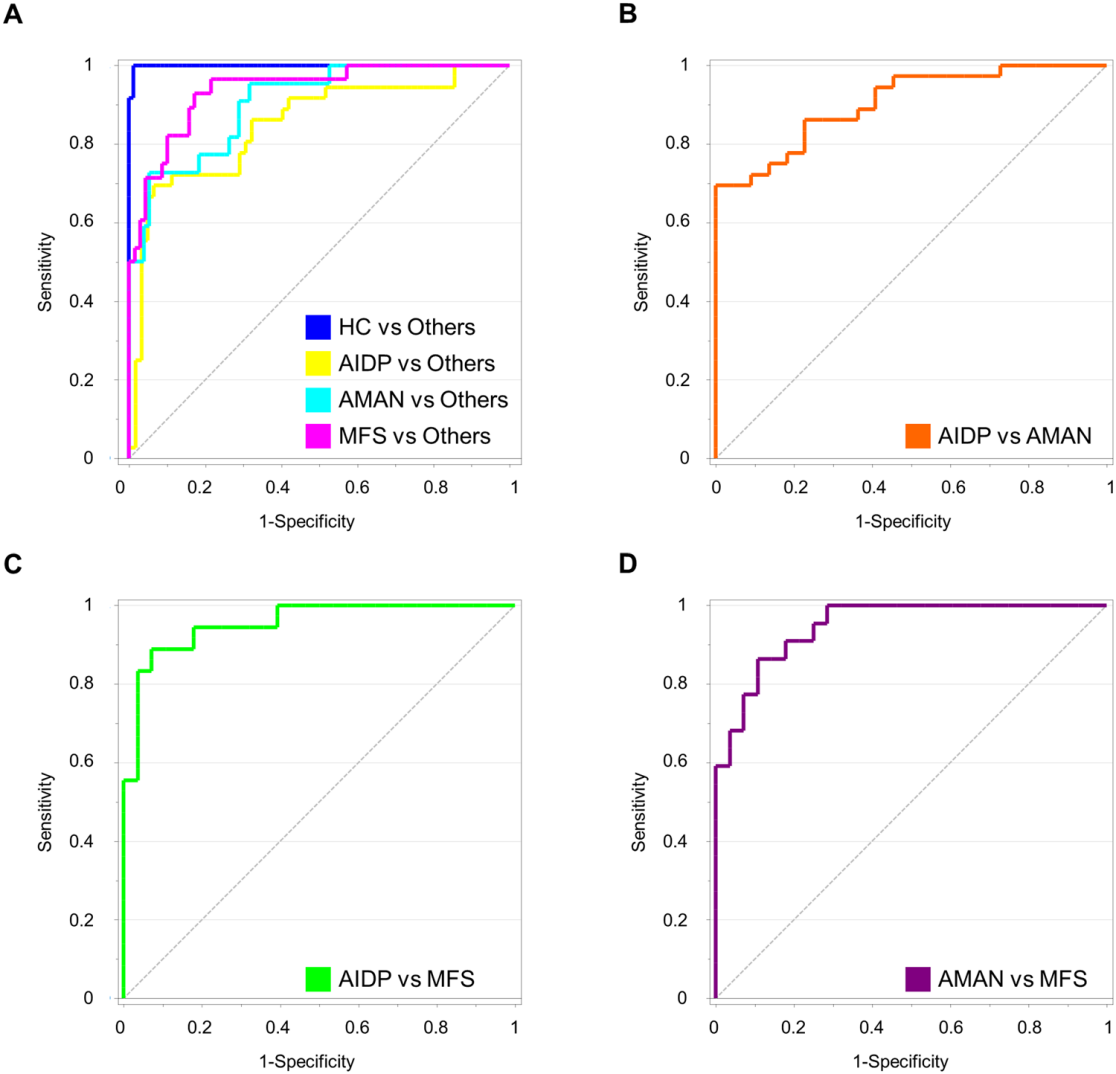
Validation of discrimination power for biomarker cluster in combination of 10 metabolites by receive operating characteristic (ROC) analysis. (**A**) ROC curves for AIDP, AMAN, MFS, and healthy controls against the other group, respectively. (**B**–**D**) ROC curves for individual group comparison among the GBS subtypes. The biomarker cluster includes: lactate, formate, acetate, glucose, 1-monopalmitin, 1-monostearin, histidine, creatinine, threose, and lysine.

Based on the model, 10 metabolites that most contributed to the discrimination were ranked by VIP analysis. Prioritized metabolites were lactate, formate, acetate, glucose, 1-monopalmitin, 1-monostearin, histidine, creatinine, threose, and lysine. The metabolites were transformed into a single value (T1 vector) by the PLS-DA, and the predictability of the metabolic signature was evaluated using ROC analysis (Fig. 4). The AUC values were significantly high for all comparison (three GBS subtypes and healthy controls). The AUC was 0.937 (95% confidence interval 0.870–0.976) for MFS against the others (AIDP, AMAN, and healthy controls). The AUCs were 0.849 (0.763–0.913) and 0.904 (0.828–0.954) for AIDP and AMAN. For additional validation of the ROC model, data set was randomly split into discovery (50%) and validation data (50%) sets. This step was repeated twice, and the result was presented in Supplementary Fig. S3. The biomarker re-composite also showed the highest levels of sensitivity and specificity against disease controls (AUC = 0.972, SN = 85.00, SP = 95.92). Likewise, the ROC curve analysis was also conducted on all pair-wise comparison between individual group. The AUCs of all three comparisons showed fairly good prediction power (Fig. 4). The AUC was 0.904 (95% confidence interval 0.798–0.966) between AIDP and AMAN. The AUCs were 0.954 (0.871–0.991, between AIDP and MFS) and 0.948 (0.846–0.991, between AMAN and MFS), respectively. Subsequently, the biomarker panel was evaluated for the discrimination power between GBS and other clinical status (healthy controls, disease controls, and IDDs). Indeed, the metabolic markers showed the highest level of predictability as proposed by the AUCs that ranged 0.978–1 (Supplementary Fig. S4). Among the ten biomarkers, lysine and histidine were unique to MFS and ADIP shared by none of the other types.

Next, we examined if the ten metabolic markers can be linearly formulated, and if the multivariate model equally performs good predictability. This allows straightforward mathematical model, and in turn more adequate clinical interpretation and application^18^. Accordingly, binary logistic regression with enter method was applied to derive a linear function based on the ten metabolic markers. Indeed, the linear algorithm showed the discriminant power equivalent to the PLS-DA model. The highest odds ratio (OR) was identified with formate (OR = 3.1, p = 0.002) from the linear algorithm (AIDP and AMAN), which resulted in the AUC of 0.962 (SN = 90.9 and SP = 91.7). Similarly, the linear combination resulted in good discrimination power between AIDP and MFS (AUC = 0.991). The linearly-recomposed metabolic marker also showed good discriminant performance (AMAN vs MFS, AUC = 0.984) (Supplementary Fig. S5).

### Characteristic phospholipid profiles of the GBS subtypes and its association with GBS disability

**Figure 5:**
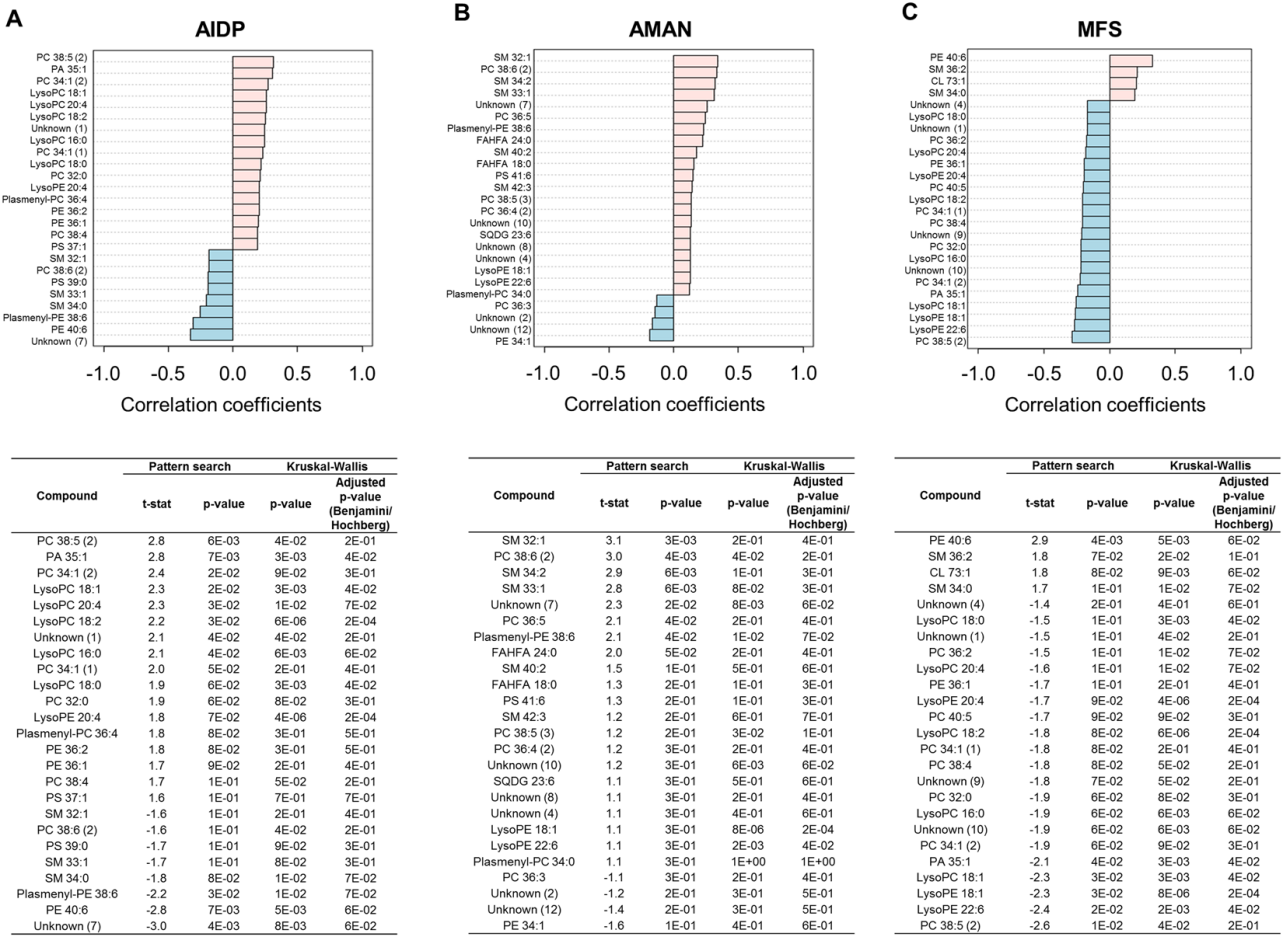
Identification of the GBS variant-specific lipid molecules. The lists included the metabolites with the highest (pink) and lowest (blue-sky) contents in AIDP (**A**), AMAN (**B**), and MFS (**C**), respectively compared to the other groups. The metabolites were selected based on Pattern Searching algorithm, which computed the fitness to pre-determined patterns in metabolite abundances based on Pearson’s correlation coefficients. For validation, analysis of variance was conducted among the three groups based on *Kruskal-Wallis* test with *Benjamini/Hochberg* adjustment. See more details in material and method.

Un-targeted lipid profiling was conducted on the GBS subtypes to characterize the differential modulation of lipid metabolism. To effectively discriminate variant-specific lipidomic changes, we applied the Pattern Searching algorithm implemented in MetaboAnalyst that identified correlation between group (the GBS variants) and variables (lipid molecules). The Pattern Searching analysis is an application of correlation analysis that captures feature or pattern that is pre-determined by numerical order according to the relative abundances by the computation of linearity, hence it identifies metabolites presenting “template matching” expression pattern (abundance) similarly with more common statistical approach, Pavlidis Template Matching (PTM)^19^. Consequently, the analysis designated the lipids showing the highest contents and the lowest contents in each GBS subtype compared to the others (Fig. 5A–C), and the results were further validated based on Kruskal-Wallis test with Benjamini-Hochberg adjustment (Fig. 5D–F). The AIDP group was mainly characterized by the highest contents of 5 lyso-phosphatidylcholines (lysoPCs) (Fig. 5A) whereas the highest contents of five SMs were unique to the AMAN group (Fig. 5B). Among others were fatty acid esters of hydroxy fatty acid (FAHFA 18:0 and FAHFA 24:0) that were specific to the AMAN group. On contrary, the lipid profiles of the MFS group were featured by the lowest contents in 21 lipids compared to other GBS variants.

**Figure 6:**
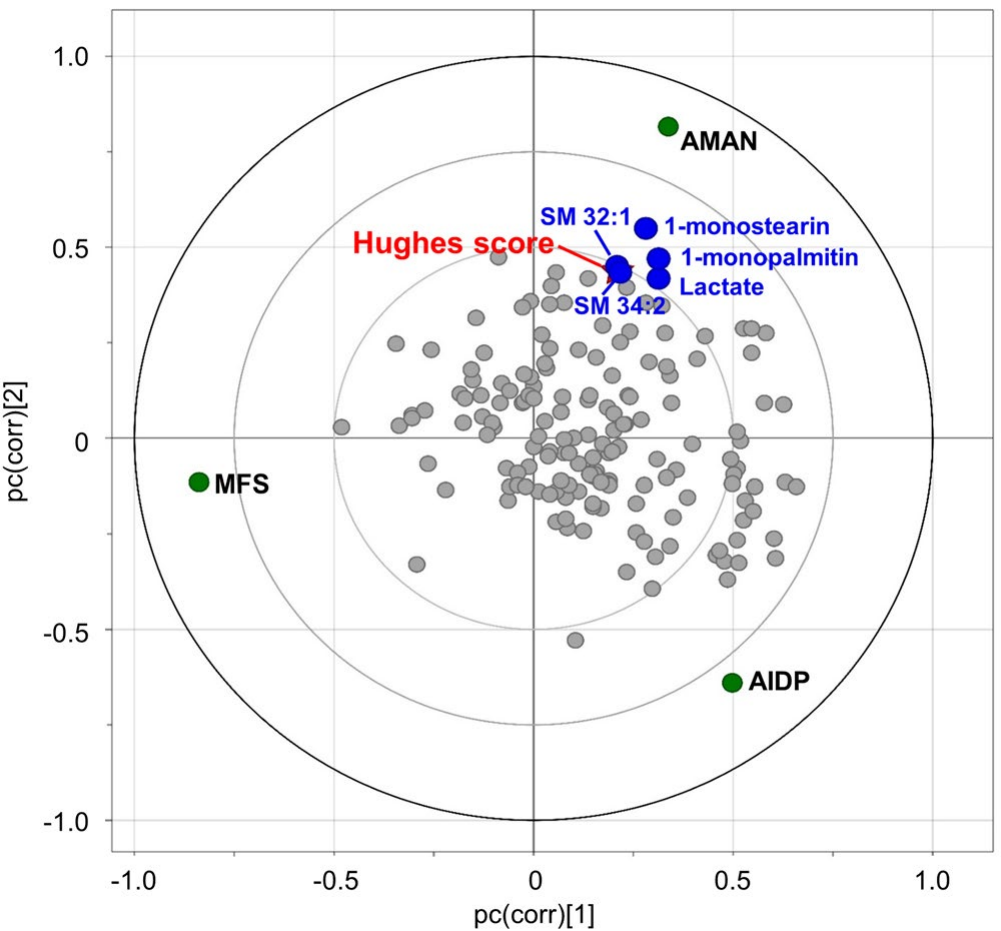
The putative correlation of CSF metabolome and GBS disability score (Hughes scoring system) among the GBS subtype. Groups and variables are mapped on loading scatter plot based on the PLS-DA model. The relative association among the GBS subtype, metabolites (primary metabolites and lipid molecules), and Hughes scores are plotted according to x-axis and y-axis, which indicate pc(corr)[1] and pc(corr)[2], respectively. Distance and direction among data points imply the level of association.

We sought a potential association of CSF metabolites with a clinical parameter, Hughes scoring system indicative of GBS disability scale. Among the GBS subtypes were the AMAN group that showed the significant linear linkage in which the distance and the direction from origin of coordination were linearly synchronized (Fig. 6). The metabolites included lactate and monoacylglycerols that were constituents of the metabolic marker panel. Others were sphingomyelins, SM 32:1 and SM 34:1. On contrary, the AIDP group presented moderate level of the association between CSF metabolome and the Hughes scale whereas the MFS group showed no correlation.

Lastly, the covarianced matrix of primary metabolites and lipids were constructed among the three GBS subtypes by Bayesian correlation network. Primarily, the network topology analysis identified potential key linkages between primary and lipid metabolism based on integrative interrogation on betweenness centrality (BC) and maximal clique centrality (MCC), which revealed the nodes with key positions in the correlation network^20^. Network topology analysis showed the greater levels of MCC in the AIDP group compared to AMAN and MFS with higher degree of connectivity (node and edge number) (Supplementary Fig. S6D). The highest level of the centralization (BC) was found in a range of primary metabolites in the AIDP group (Supplementary Fig. S6A). It included acetone, a ketone body produced during ketoacidosis. Others were acetate, citrate, lactate, amino acids (glutamine, leucine, and threonine), and free fatty acids (myristic acid, palmitic acid, heptadecanoic acid, and stearic acid). Among lipids, lysoPCs (C18:0 and C18:1) were highly centralized in the AIDP group. The key modules in MFS network were identified in such primary metabolites as acetate, benzoic acid, octadecanol, heptadecanoic acid, nitrogenous compounds (alanine, valine, ornithine, creatinine, and choline), and xylose (Supplementary Fig. S6C). On contrary, the AMAN network presented the lowest levels of both centralities (BC and MCC) with relative scarcity in the connectivity (Supplementary Fig. S6B).

## Discussion

Identification of biomarkers and patho-mechanistic understanding of GBS is critical given that early and precise diagnosis helps proper treatment and prognostic procedure, which may significantly reduce severe disease course, pain, and residual deficits^21^. Diagnosis by clinical judgement in combination with examination on anti-ganglioside antibodies is possible^2,5^, but clear clinical decision cannot be always achieved, in particular for young patients in early stage^22,23^.

Accordingly, high-throughput molecular profiling such as metabolomic analysis can be an attractive and complementary option for biomarker discovery. More importantly, metabolomics can generate working-hypothesis, which allows the discovery of novel patho-mechanisms of disease without prior knowledge^24^. Accordingly, our primary goal was to metabolically characterize the GBS subtypes associated with different pathological mechanisms (e.g. AIDP vs AMAN) and targets (e.g. peripheral nerve vs cranial nerve). At the same time, we extended our investigation to defining universal properties between different autoimmune diseases (e.g. inflammatory disorder of central nerves Vs peripheral nerves), which has not been previously reported.

Here, we reported the most comprehensive study on the CSF metabolome of common clinical subtypes of GBS (AIDP, AMAN, and MFS) by applying and integrating analytical approaches, GC-MS, LC-MS, and NMR. For exclusively identifying and evaluating disease-specific metabolites, a comparative analysis was performed based on multiple baselines (healthy controls, disease controls, and samples from patients with inflammatory disorders of central nervous system). The comprehensive statistical validation characterized unique metabolic features of GBS, and consequently proposed robust biomarker panel. Moreover, metabolome-wide multivariate correlation analysis determined hypothetical linkage between disability scale (GBS Hughes score) and CSF metabolites (lactate, 1-monopalmitin, 1-monostearin, and sphingomyelins), particularly in the AMAN group. It should be noted the singular value derived from 10-metabolite recomposite, which were multivariate-statistically transformed or linearly re-formulated, allowed simultaneous discrimination of all different immune statuses in different comparison combination. The robustness may come from the highest level of data quality (e.g. confirmative identification process in combination with manual inspection), and systematic and unbiased feature selection although it led to the relatively smaller number of identified metabolite in our study.

There have been investigations on the syndrome based on high-throughput molecular profiling by proteomic and microarray analyses that suggested potential molecular mechanisms and biomarkers for GBS based on CSF and blood samples^9–11^. More recently, an untargeted metabolomic approach proposed a disturbance of lipid metabolism and molecular indicators for the syndrome and its variants based on blood plasma^25^. However, CSF metabolomic investigation on GBS and its variants has not yet been reported. To analyze CSF metabolome is important because CSF interfaces directly with myelin sheath and nerve exon that are primarily damaged by post-infectious autoimmune response. Accordingly, we identified a unique metabolic phenotype that distinguishes GBS from healthy controls, disease controls, and IDDs (MS and NMOSD).

The metabolic distinctiveness was featured by the alteration of glucose and acetoacetate. The elevated levels of glucose and the ketone body are a typical clinical symptom in diabetics. Hyperglycemia and ketoacidosis have been reported in blood of GBS patients^26–29^, but it has not been reported that this clinical finding also manifests in the CSF metabolome. On contrary, decreased glucose content was observed in IDDs. Likewise, lactate has been proposed as a common attributor to a range of diseases including inflammation^12^; however, the significantly lower level of lactate was found in GBS but not in IDDs in our experiment. Acetate is a precursor for myelin lipid biosynthesis in neurolemmocyte and oligodendrocytes^30^. Consistent with our result, the reduced level of acetate suggested the abnormality of myelin lipid biosynthesis possibly caused by aspartoacylase dys-function (ASAP)^31–33^.

In addition, the inflammatory diseases of peripheral and central nervous systems shared common metabolic disorder. For instance, the higher level of glycolic acid was common feature for both GBS and IDDs. We also identified common metabolic dys-regulation in all disease status (GBS, IDDs, and disease controls). The metabolite list included threose, methionine, inosine, 1-monopalmitin, and 1-monostearin. The uncommon monoacylglycerols (1-monopalmitin and 1-monostearin) were present at the elevated levels, which may be involved in common pathogenesis among neurological illness^12^.

Next, we mined unique biochemical trait and biomarker signature focusing on the GBS subtypes. It has been suggested that the pathological mechanism was similar between AMAN and MFS (e.g. Anti-ganglioside antibodies attack nerve axon primarily.)^34,35^ whereas AIDP and AMAN shared common clinical symptom (e.g. limb motor weakness)^2,36^. The primary metabolic profiles coincided the comparable clinical symptom between AIDP and AMAN while the MFS was clearly distinctive from the two subtypes. First, the hypermetabolic activity was characteristic to the MFS group in which CSF glucose was present at significantly higher levels compared to healthy controls. A previous report on the MFS involvement with hypermetabolism in the central nervous system suggested GQ1b associated acute inflammatory process as a mechanism of this autoimmune disorder^37^. The differential levels of acetoacetate, creatinine^7,25,38^, and pyruvate^39^ further supported the hypermetabolic activity of MFS. In addition, the reduced levels of proteinogenic amino acids (leucine, lysine, isoleucine, phenylalanine, alanine, and proline) may correspond to elevated levels of CSF protein in most of the patients with MFS^40^.

On contrary, differential regulation in saturated fatty acid (SFA) metabolism was identified in AIDP and AMAN. The both subtypes presented the elevated level of stearic acid (C18:0). AIDP presented additional changes in myristic acid and palmitic acid whereas AMAN showed increased levels of lauric acid and heptadecanoic acid. The increased pool sizes in the fatty acids may correspond to a recent report on depleted levels in a range of phospholipids and sphingomyelin, indicative of more significant dysfunction of myelin lipid biosynthesis or severer demyelination process in AIDP and AMAN relative to MFS^41^. Based on our findings, we proposed a biomarker model that allowed simultaneous discrimination of individual subtype from each other, and also healthy and disease controls. The robustness of the biomarker panel was further proved by different combination of statistical validation. For instance, the comparison by pair-wise discrimination between individual group (e.g. AIDP vs AMAN) also showed the equivalent levels of prediction power. Besides, binary logistic regression analysis successfully re-formulated linear functions with the metabolic markers, which may lead to more direct clinical application via simple mathematical model. The metabolites panel composed of 10 CSF metabolites represented quantitative distinctiveness, and implied putative biochemical linkage to the pathology. The compositional differences were characterized as follows: Monoacylglycerols (1-monopalmitin and 1-monostearin), threose, methionine, and inosine contributed to the discrimination of all disease groups from healthy. The depleted contents of creatine and lysine were specific to MFS whereas aberrant level of histidine was exclusively observed in AIDP. The metabolites of major energy metabolism, glucose and lactate were common feature in AIDP and MFS.

We further investigated on lipidomic profiles focusing on the GBS subtypes. Phospholipids (PLs) are important components of biological membranes and precursors of numerous signaling molecules^42^. Neurological dysfunction in GBS patients is attributed as reduced conductance associated with demyelination and/or axonal loss, which couples with membrane lipid composition. The CSF lipidome of AMAN was primarily characterized by significantly higher levels in SMs, indicative of accelerated loss in myelin structure^43^, which was not observed in the AIDP and MFS groups. Interestingly, the elevated FAHFAs was found unique to patients with AMAN. FAHFAs, formed in adipose tissue, have shown anti-inflammatory activity and glucose tolerance improvement^44,45^. The over-production of the endogenous lipid may represent the distinctive protective mechanism in AMAN or may play a pivotal role like arachidonic acid that is metabolized to both proinflammatory and anti-inflammatory eicosanoids during and after physical activity to promote growth^46^. The AIDP group was characterized by the highest contents of lysoPCs compared to other GBS variants. The elevated levels of lysoPCs have been proposed as a potent demyelination reagent via rapid influx of T cells^47^.

Moreover, we identified potential clinical association of the CSF metabolome. The highly-coordinated distribution was determined among the AMAN group, Hughes score, and metabolites (lactate, 1-monopalmitin, 1-monostearin, SM 32:1, and SM 34:1). The strongest linearity in the AMAN group was plausible considering higher levels in Hughes score compared to the AIDP group, and differential disability mechanism in the MFS group. Of particular interest, the monoacylglycerols have been consecutively implicated in dysregulated immune system^48^ and related disabling symptom^12,49^. Likewise, elevated level in CSF lactate has been reported for acute attack in neuromyelitis optica spectrum disorder (NMOSD) during remission^50^.

Finally, network topological analysis suggested key metabolic modules, indicative of potentially important role in biochemical network^51^. For instance, metabolites in cliques (clusters) are under identical relation with all other members, which may indicate their synchronous co-regulation to the remaining metabolic modules^20^. Particularly, metabolites connecting between clusters may function as branch points of known or unknown biochemical linkage or paths bridging different metabolic cycles^20^. Indeed, our result showed palmitic acid, identified as unique metabolic feature to AIDP, showed high centrality in both betweenness and maximal clique. The most abundant circulating fatty acid has been suggested as system activator in immune system^52^. Also, stearic acid was identified as a key module with significant increase in the AIDP group. The distinct association with a broad range of lipid metabolism in AIDP was further remarked by lysoPCs (C18:0 and C18:1) that also showed the AIDP-specific expression pattern. In addition, acetone, a byproduct of ketoacidosis, was shown high centrality and connectivity in the subtype. And β-hydroxybutric acid, a ketone body confirmatively implied aberrant metabolic activity in the AIDP group. On contrary, creatinine, ornithine, and cardiolipin, which of all were also exclusively dys-regulated in the MFS group, were found highly centralized in the network. Abnormal expression levels in creatinine and ornithine may reflect dysfunction of creatine kinase, which has been associated with GBS^7,53^. Creatine, found in significantly reduced contents across all GBS variants, has been suggested as the hallmark of mitochondrial dysfunction^54,55^, which was further inferred from dys-regulation in cardiolipin, a major constituent of the mitochondria inner-membrane. In addition, anti-cardiolipin antibodies have been reported in the association with autoimmune diseases^56^.

There are some limitations of our study. GBS is a rare autoimmune disorder, thus our exploratory conclusion was limited by relatively small study size, which requires the expansion of observation and additional examination for the validity. In addition, the syndrome involves acute pathological progress and requires such immediate and non-specific therapies as intravenous immunoglobulin or plasma exchange. The circumstances hindered long-term and subsequent examination (e.g. prognostic application). Besides, CSF sampling is generally accompanied by invasive procedure (lumbar puncture), and the amount of the samples is not sufficient for a range of baseline test. It may cause limited availability of some clinical data, contrast to blood sampling, which is relatively feasible to acquire (e.g. white blood cell count, protein contents, and cholesterol contents).

Nonetheless, to our knowledge, this is the first and the most comprehensive study on integrative profiles of primary metabolites and lipids from CSF samples with GBS patients. We performed the exclusive evaluation based on multiple baseline (healthy controls, disease controls) and comparative analysis with inflammatory disorder of central nervous system (e.g. IDDs). The integrative metabolomic approach systematically characterized metabolic dysfunction reflected in the CSF of the GBS subtypes. The unique metabolic features of primary metabolic dysfunction and aberrant lipid metabolism may lead to better understanding, and to discovery of unique biomarker signature and new therapeutic strategy for future clinical application.

## Methods

### Patient information and clinical manifestations

CSF samples were obtained from the patients with AIDP, AMAN, MFS, and disease controls during the acute stage within 2 weeks from onset of symptom but before any specific medication or treatment. The collection was done by Dong-A University Neuroimmunology Team for anti-ganglioside antibody study (from 2010 to 2015). CSF samples were consecutively collected from the patients who were suspected to have IDDs by National Cancer Center registry for inflammatory diseases of the CNS from May 2005 to April 2015. The detailed information on demographic and clinical data of healthy controls and the IDD patients were available from our previous report^12^.

CSF samples from 86 patients were analyzed for diagnosis of GBS subtyping. CSF samples of disease controls were obtained from 20 cases who underwent lumbar puncture to rule out autoimmune neuropathy or meningitis vice versa, but turned out to have other neurological illness than GBS or associated variants. All CSF samples were stored at −80 °C until analysis. The demographic and clinical data of the patients, including age, gender, dates of sampling, and GBS subtype, were retrospectively collected. Diagnosis of GBS was based on the current criteria^57^ and each subtype was determined by the electrophysiological, immunological and clinical information by each referring specialists. The AMAN and MFS groups were further classified by positive anti-ganglioside body (anti-GM1 and anti-GQ1b antibodies in each group) for more accurate classification. The patients with exclusive anti-GM1 positive outcome were included in the AMAN group whereas the patients with exclusive anti-GQ1b positive result were categorized as the MFS group in the current study. Hughes score was categorized as follows: 0, A healthy state; 1, Minor symptoms and capable of running; 2, Able to walk 10 m or more without assistance but unable to run; 3, Able to walk 10 m across an open space with help; 4, Bedridden or chair bound; 5, Requiring assisted ventilation for at least part of the day; 6, Dead^58^.

This study was approved by the research ethics committee of Dong-A University Hospital (No. 10-10-7). All the procedures were carried out in accordance with the Institutional Review Board of Dong-A University Hospital, and written informed consent was obtained from all subjects.

### Extraction

The extraction, derivatization, mass-spectrometry analysis, and NMR spectroscopic analysis were performed in random order on all samples. CSF samples were thawed on ice at 4 °C and 100 µL CSF sample was aliquoted and extracted with 650 µL of extraction solvent (methanol:isopropanol:water, 3:3:2, v/v/v). The mixture was sonicated for 10 minutes and centrifuged at 13,200 rpm for 5 minutes at 4 °C. Each supernatant (700 µL) was transferred into a new 1.5-mL tube. The aliquots were concentrated and completely dried in a speed vacuum concentrator (SCANVAC, Korea). The dry extracts were stored at −80 °C until derivatization and gas chromatography time-of-flight mass spectrometry (GC-TOF MS) analysis.

For NMR analysis, 400 μl of CSF sample was mixed with 100 μl stock solution of NMR buffer containing 550 mM sodium phosphate buffer. The final NMR samples contained 100 mM sodium phosphate buffer (pH 7.0), 2 mM of trimethylsilyl-propanoic acid (TSP), and 10% D_2_O.

For lipid profiling, the extraction procedure was conducted based on the Folch method with minor modification^59,60^. 250 *μ*L of CSF sample was mixed with 225 *μ*L MeOH and vortexed for 10 s. 450 *μ*L chloroform (CHCl_3_) was then mixed and incubated for 1 hr. Phase separation was done by adding 187.5 *μ*L of H_2_O. The lower phase was transferred to new vial, dried, and stored until MS analysis.

### Derivatization for GC-TOF MS analysis

The dried extracts were mixed with 5 µL of 40 mg/mL methoxyamine hydrochloride (Sigma-Aldrich, St. Louis, MO, USA) in pyridine (Thermo, USA) and then incubated for 90 min at 200 rpm and 30 °C for methoxyamination. After the first derivatization process, 2 µL of a mixture of internal retention index (fatty acid methyl esters [FAMEs]) and 45 µL of *N*-methyl-*N*-trimethylsilyltrifluoroacetamide (MSTFA + 1% TMCS; Thermo, USA) were added for trimethylsilylation (1 h at 200 rpm and 37 °C). The FAME mixture was composed of C8, C9, C10, C12, C14, C16, C18, C20, C22, C24, C26, C28, and C30.

### GC-TOF MS analysis

The derivatives (0.5 µL) were injected using an Agilent 7693 ALS (Agilent Technologies, Wilmington, DE, USA) in splitless mode. Chromatographic separation was carried out using an Agilent 7890B gas chromatograph (Agilent Technologies) equipped with an RTX-5Sil MS column (Restek, Gellefonte, PA, USA). The oven temperatures were programmed at 50 °C for 1 min, ramped at 20 °C/min to 330 °C, and held constant for 5 min. Mass spectrometry analysis was performed on a Leco Pegasus HT time of flight mass spectrometer controlled by ChromaTOF software 4.50 version (LECO, St. Joseph, MI, USA). Mass spectra were acquired in the mass range of 85–500 *m/z* at an acquisition rate of 20 spectra/s.

Using ChromaTOF software, data including apex mass, full spectrum, retention time, signal-to-noise ratio, and peak purity, were exported to our own server computer. Pegasus files (.peg) were converted to text files (.txt) and netCDF files for further data evaluation. Based on *Binbase* algorithm, raw data files (text files) were processed using the criteria as follows; a) <10 peaks with intensity >10^7^ counts s^−1^ for chromatogram validation, b) spectra similarity >800 for RI detection, c) 5th order polynomial regression-based retention index calculation, d) retention time window of ±2 s, e) validation of unique mass against apex mass^18,61^. A quantification data based on peak height of single quant ion was generated for database entries (Fiehn library and NIST library). Missing values that did not pass the primary criteria were imputed by post-matching and replacement using raw data as previously described^62^. To check the analytical stability, a mixture of 19 pure reference compounds was analyzed every 10 samples. The data for the quality control is provided in Supplementary Fig. S7A.

### UPLC-Orbitrap MS analysis

The dried samples were re-constituted with 50 µl of 70% acetonitrile for lipid profiling. The samples were chromatographically separated with a 150 × 2.1 mm UPLC BEH 1.7-μm C18 column (Waters, USA) equipped with 5.0 mm × 2.1 mm UPLC BEH 1.7 μm C18 VanGuard Pre-Column (Waters, USA) controlled by Ultimate-3000 UPLC system (Thermo Fisher Scientific, USA). The mobile phase was 10 mM ammonium formate, 0.2% formic acid in water (buffer A) and 0.2% formic acid in acetonitrile (buffer B). LC condition was as follows: equilibration in 10% buffer B for 1 min, 10–75% buffer B gradient over 7.5 min, 75–95% buffer B gradient over 7.6 min, 95% buffer B for 2.8 min and re-equilibration in 10% buffer B for 5.5 min. Injection volume of sample was 10 µl for both full scan (MS1) and MS/MS analysis. Mass spectra were acquisition in negative ionization using Q-Exactive Plus Orbitrap (Thermo Fisher Scientific, USA). Data-dependent MS/MS analysis were conducted with 14 samples for first collecting the list of putatively annotated lipids (collision energy, 30 eV) against the Lipid blast^63^ and Lipid search library (Thermo Fisher Scientific, USA) in which relatively lower threshold (MS1 tolerance, 0.005 Da; MS2, 0.05 Da) was set to reduce false negative discovery rate. A total of 89 retention time-exact mass pairs (i.e. features) were putatively annotated, and further considered for quantitative evaluation after redundancy and peak quality check using Tracer Finder (Thermo Fisher Scientific, USA) (Supplementary dataset 1). Among them, the identification of 57 lipids were validated by manually inspecting MS/MS spectra.

### NMR spectra acquisition and analysis

To analyze metabolites, one-dimensional ^1^H-NOESY NMR spectrum was obtained at 298 K on a Bruker ASCEND III 600 spectrometer equipped with a cryoprobe. The NOESY pulse sequence (noesygppr1d) was applied with presaturation to suppress the residual water signal. ^1^H-NMR spectrum for each sample consisted of 256 scans with following parameters: spectral width = 12,019.2 Hz, spectral size = 65,536 points, pulse width (90) = 12.2 μs, relaxation delay (RD) = 5.0 s, and a mixing time of 10 ms.

For quantitative profiling of CSF samples, spectra were processed and analyzed with Bruker topspin 3.1 (Bruker GmbH, Karlsruhe, Germany) and Chenomx NMR suite 7.7 (Chenomx Inc., Edmonton, Canada). Each free induction decay (FID) was zero-filled to 64,000 points and transformed with line broadening (LB) = 0.3 Hz. NMR spectra were initially phased in Bruker topspin 3.1 and the final baseline was corrected using Chenomx NMR suite 7.7. The baseline model was built in each spectrum using the algorithm of multipoint baseline correction. The chemical shifts were referenced against the resonance of TSP as 0.0 ppm. Metabolites were identified using the database stored in Chenomx NMR suite 7.7 and were quantified from the comparison of the internal standard (TSP). A total of 31 metabolites identified from the CSF samples. The identified metabolites were evaluated in ^1^H-^13^C HSQC and 2D ^1^H-TOCSY spectra.

### Data transformation and batch effect removal

The number of metabolites identified from two studies was summarized as follows: 83 and 31 metabolites were identified from the GBS metabolic profiles by GC-TOF MS and NMR, respectively. Likewise, 85 and 32 metabolites were acquired from IDDs study (Supplementary Fig. S8A). A total of 100 metabolites were commonly detected in both studies. For constructing compatible data matrix acquired from GC-TOF MS, the batch effects between the GBS study and published data (IDDs and healthy controls) from previous study^12^ were removed using COMBAT algorithm (SVA package) in R environment (version 3.1.3). Primary PCA scores matrix (P1) were computed for numericalizing the expression values of integrative metabolite panels. The PCA scatter plot proved that data normalization successfully removed the batch effect (principal component 1), so they were directly comparable (Supplementary Fig. S8B,C).

### Statistical analysis

Statistical analyses were conducted on all continuous variables collected from GC-TOF MS, LC-MS, and NMR. The metabolites detected both in GC-TOF MS and NMR were further speculated. In general, the relative abundances were comparable between the two analytical platforms (Supplementary Fig. S9). The ones analyzed by NMR were selected for further statistical analysis since NMR is more adequate for quantitative analysis^64^ and the reproducibility is better in NMR (Supplementary Fig. S9). After removing duplicate metabolites between GC-TOF MS and NMR, a total of 87 metabolites among primary metabolites were confirmatively identified and further used for normalization. Processed data was normalized by the sum of identified peaks (GC-TOF MS), the sum of selected features of each chromatogram (LC-MS) within each group^65^, or the sum of quantification values (NMR) across all groups (Supplementary dataset 1 and 2). The raw and processed data are available at the website (https://lms2.kookmin.ac.kr:446/index.php?hCode=PAPER_LIST&publication_name=inter_paper). *P*-values were calculated by the two tailed Student’s T-test in Excel (Microsoft Office 2016) and one-way analysis of variance was conducted based on *Kruskal-Wallis* test with *Benjamini*-*Hochberg* adjustment using *Multi Experimental Viewer* (MeV, TIGR)^12,66^. Box-and-whisker plots were generated using MetaboAnalyst 4.0 (http://metaboanalyst.ca).

Principal component analysis (PCA), partial least squares-discriminant analysis (PLS-DA), variable importance in projection (VIP), and Receiver Operating Characteristic (ROC) analysis were done using SIMCA 14 (Umetrics AB, Umea, Sweden). PLS-DA was performed using 7-fold cross-validation and permutation test (N = 999). For validation of ROC analysis, the 95% confidence interval using bootstrapping was done by biomarker analysis module implemented in Medcalc® (Medcalc® Software, Mariakerke, Belgium). Multivariate analysis of covariance (MANCOVA), and binary logistic regression analysis with enter method were performed by IMB SPSS Statistics for Windows, version 23.0 (IBM Corp., Armonk, N.Y., USA).

Pathway enrichment analysis was performed using MetaboAnalyst 4.0 (http://metaboanalyst.ca)^67^. Statistical significance was evaluated based on the hypergeometric test and pathway topology was analyzed based on the relative-betweenness centrality. Pattern Searching module implemented in MetaboAnalyst 4.0 was applied to pre-determine the lipids with the disease type-specificity. The pattern is pre-defined as consecutive number. For instance, that can be formulated as 2-1-1 where the position of each digit indicate pre-assigned group (MFS-AMAN-AIDP) while each value presents relative abundances among the three group (in this case, metabolites with the highest contents in MFS and with lower contents in AMAN and AIDP). The univariate statistical analysis validated based on Kruskal-Wallis test with Benjamini-Hochberg adjustment (*Multi Experimental Viewer*, TIGR).

For primary metabolite-lipid network construction, linear relationships were computed based on the Bayesian law and Hess normal form^68^. The correlation matrix was then imported to Cytoscape interface^69^ and cytoHubba plugin^20^ for further visualization and network topological analysis. Subnetworks were finally visualized with top 20-ranked nodes by maximal clique centrality and their edges.

### Quality control for mass spectrometric analysis

Extraction, derivatization and mass spectrometric analysis were randomly conducted to reduce any potential systematic error. In addition, analytical stability of mass spectrometry was evaluated prior to data analysis. The quality control (QC) mixture consisting of 19 representative metabolites, was analyzed every ten samples. The percentage of coefficient of variation (%CV) was 14.9 across 8 QC samples. Score scatter plot of the QC mixture computed by PCA showed the constant levels of the compounds throughout the analysis (Supplementary Fig. S7A). In addition, orthogonal projection in latent structure (OPLS) analysis was conducted to globally interrogate potential effect of sample storage period on CSF metabolome as confounding effect. The result indicated no significant correlation between the storage period and CSF metabolites (pq < 0.3) (Supplementary Fig. S7B).

## Supplementary information

Supplementary material

Supplementary data 1

Supplementary data 2
