# Supplementary material for: Integrative metabolomics reveals unique metabolic traits in Guillain-Barré Syndrome and its variants
Source: Sci Rep. 2019 Jan 31;9:1077. doi: 10.1038/s41598-018-37572-w (PMC6355784; doi:10.1038/s41598-018-37572-w)
Supplement: Supplementary file 1 — Supplementary material [file 41598_2018_37572_MOESM1_ESM.docx]

**Integrative metabolomics reveals unique metabolic traits in Guillain-Barré Syndrome and its variants**

Soo Jin Park^1¶^, Jong Kuk Kim^2¶^, Hyun-Hwi Kim^3^, Byeol-A Yoon^2^, Dong Yoon Ji^1^, Chang-Wan Lee^1^, Ho Jin Kim^4^, Kyoung Heon Kim^5^, Ha Young Shin^6^, Sung Jean Park^3*^, Do Yup Lee^1*^

^1^ The Department of Bio and Fermentation Convergence Technology, BK21 PLUS Program, Kookmin University, Seoul 02707, Republic of Korea

^2^ Department of Neurology, Peripheral Neuropathy Research Center, Dong-A University College of Medicine, Busan, 49315, Republic of Korea

^3^ College of Pharmacy and Gachon Institute of Pharmaceutical Sciences, Gachon University, Incheon 21936, Republic of Korea

^4^ The Department of Neurology, Research Institute and Hospital of the National Cancer Center, Goyang, Republic of Korea

^5^ The Department of Biotechnology, Graduate School, Korea University, Seoul, Republic of Korea

^6^ Department of Neurology, Brain Korea 21 Project for Medical Science, Yonsei University College of Medicine, Seoul, Korea

^¶^These authors contributed equally to this work

^*^Corresponding Authors:

Prof. Do Yup Lee: Tel: + 82-2-910-5733; Email: [rome73@kookmin.ac.kr](mailto:rome73@kookmin.ac.kr)

Prof. Sung Jean Park: Tel: +82-32-899-6113; Email: psjnmr@gachon.ac.kr

Soo Jin Park: [sj_1307@naver.com](mailto:sj_1307@naver.com)

Jong Kuk Kim: [advania9@chol.com](mailto:advania9@chol.com)

Hyun-Hwi Kim: [hyunhwikim@gmail.com](mailto:hyunhwikim@gmail.com)

Byeol-A Yoon: [yoonstara@nate.com](mailto:yoonstara@nate.com)

Dong Yoon Ji: [jdy5668@nate.com](mailto:jdy5668@nate.com)

Chang-Wan Lee: [lca0133@gmail.com](mailto:lca0133@gmail.com)

Ho Jin Kim: [hojin@ncc.re.kr](mailto:hojin@ncc.re.kr)

Kyoung Heon Kim: [khekim@korea.ac.kr](mailto:khekim@korea.ac.kr)

Ha Young Shin: [HAYSHIN@yuhs.ac](mailto:HAYSHIN@yuhs.ac)

Young-Eun Park: [yepark@pusan.ac.kr](mailto:yepark@pusan.ac.kr)

Eun Hee Sohn: [Seh337@hanmail.net](mailto:Seh337@hanmail.net)

Min Su Park: [minsupark@ynu.ac.kr](mailto:minsupark@ynu.ac.kr)

Dae-Seong Kim: [dskim@pusan.ac.kr](mailto:dskim@pusan.ac.kr)

Sung Jean Park: [psjnmr@gachon.ac.kr](mailto:psjnmr@gachon.ac.kr)

Do Yup Lee: [rome73@kookmin.ac.kr](mailto:rome73@kookmin.ac.kr)

**Supplementary figure**


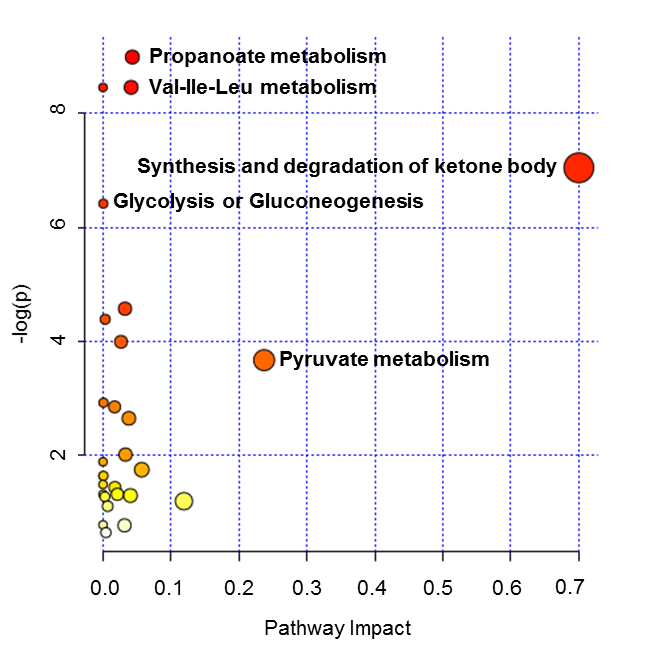


**Fig S1. Pathway over-representation analysis of GBS against healthy controls.** The list included the metabolites with statistical significance (p < 0.05). X-axis is pathway impact values, computed based on the central betweenness of a node (a metabolite). Y-axis presents the significant level. For visual clarification, the pathway impact and statistical significance are visualized in proportion to node radius and color, respectively.

**
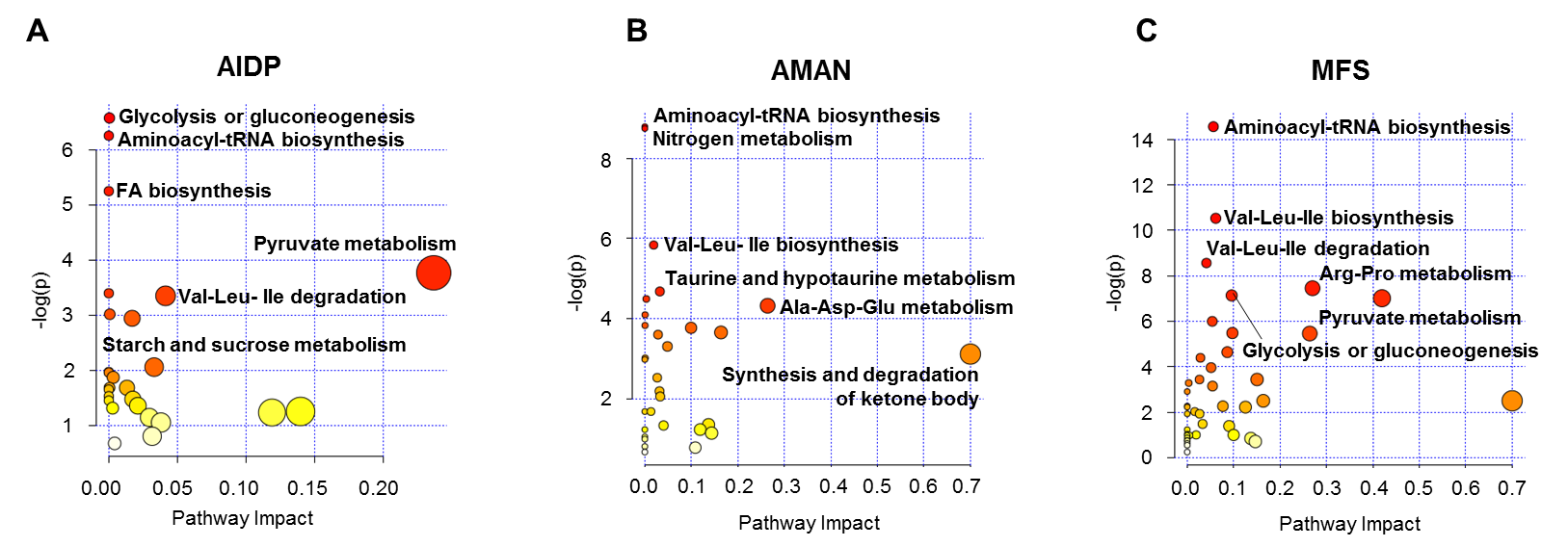
**

**Fig S2. Pathway over-representation analysis of GBS subtypes against healthy controls.** The list included the metabolites with statistical significance (p < 0.05). X-axis is pathway impact values, computed based on the central betweenness of a node (a metabolite). Y-axis presents the significant level. For visual clarification, the pathway impact and statistical significance are visualized in proportion to node radius and color, respectively.


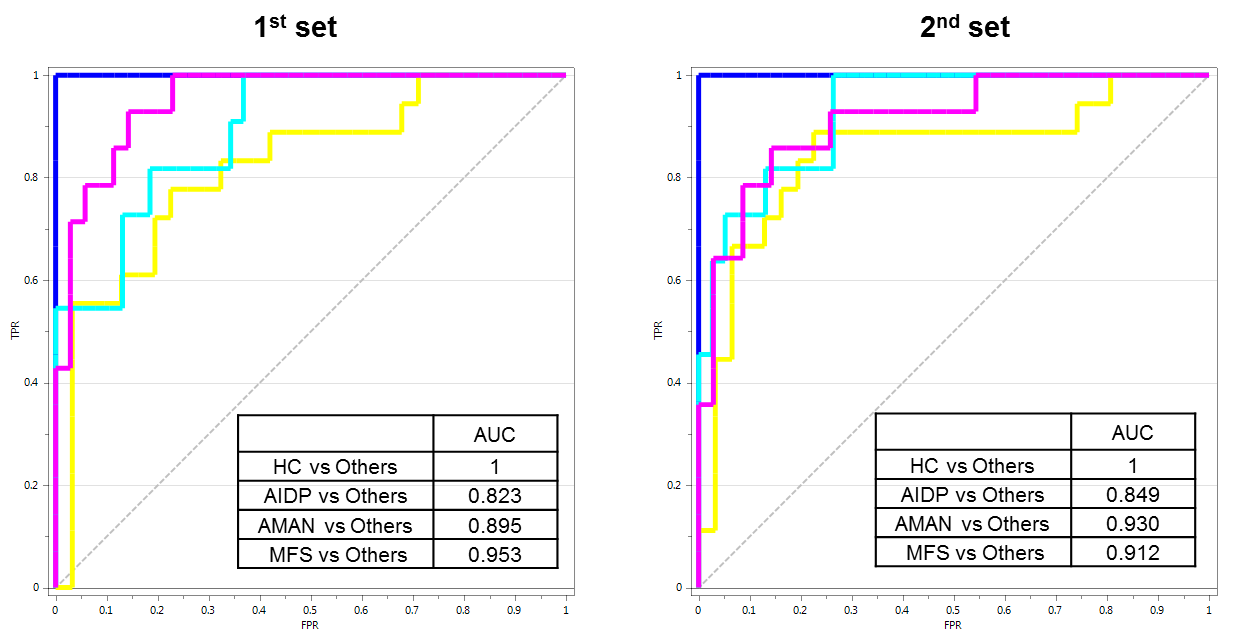


**Fig S3. Receiver operating characteristic (ROC) curve analysis of two randomly-selected subgroups for additional inter-validation.**


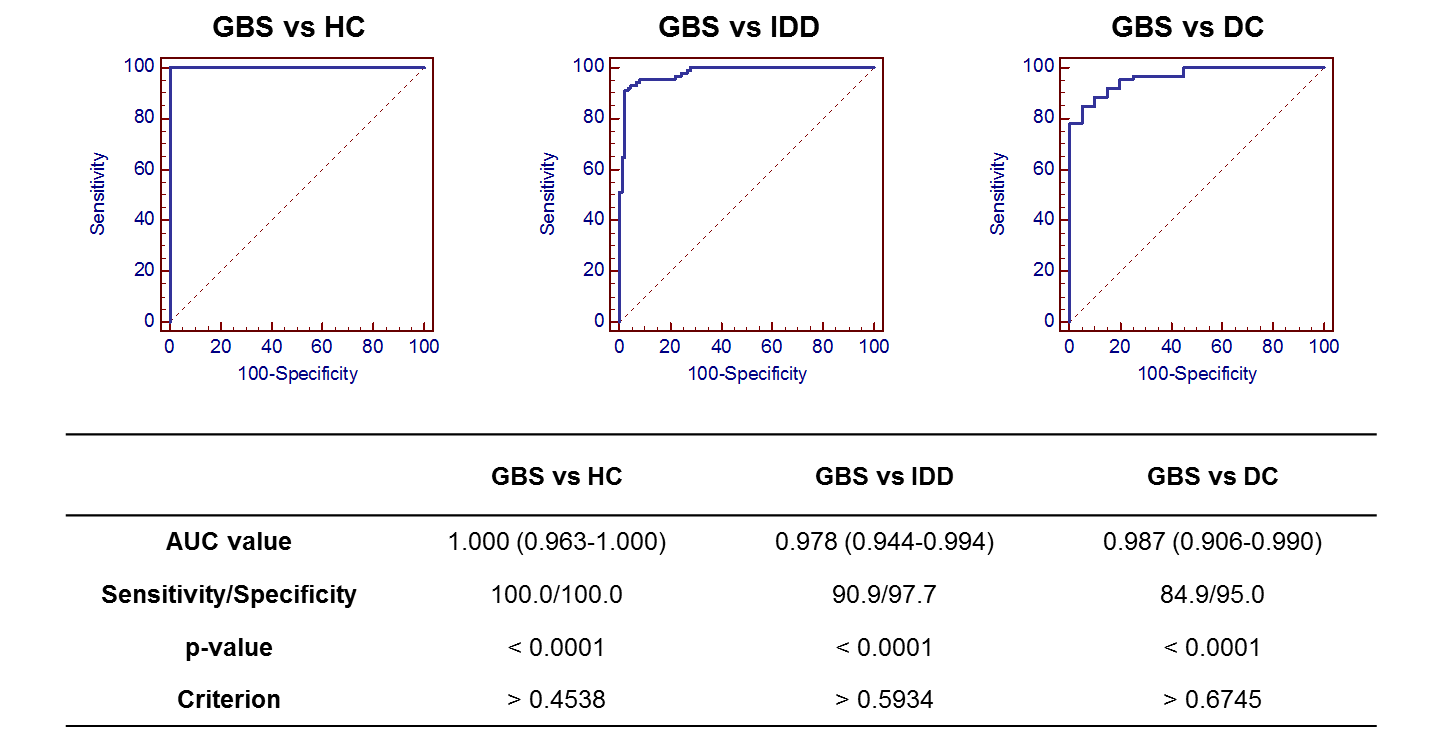


**Fig S4. Receiver operating characteristic (ROC) curve analysis of GBS against healthy controls, IDDs, and disease controls with identical list of metabolite markers (lactate, formate, acetate, glucose, 1-monopalmitin, 1-monostearin, histidine, creatinine, threose, and lysine.**


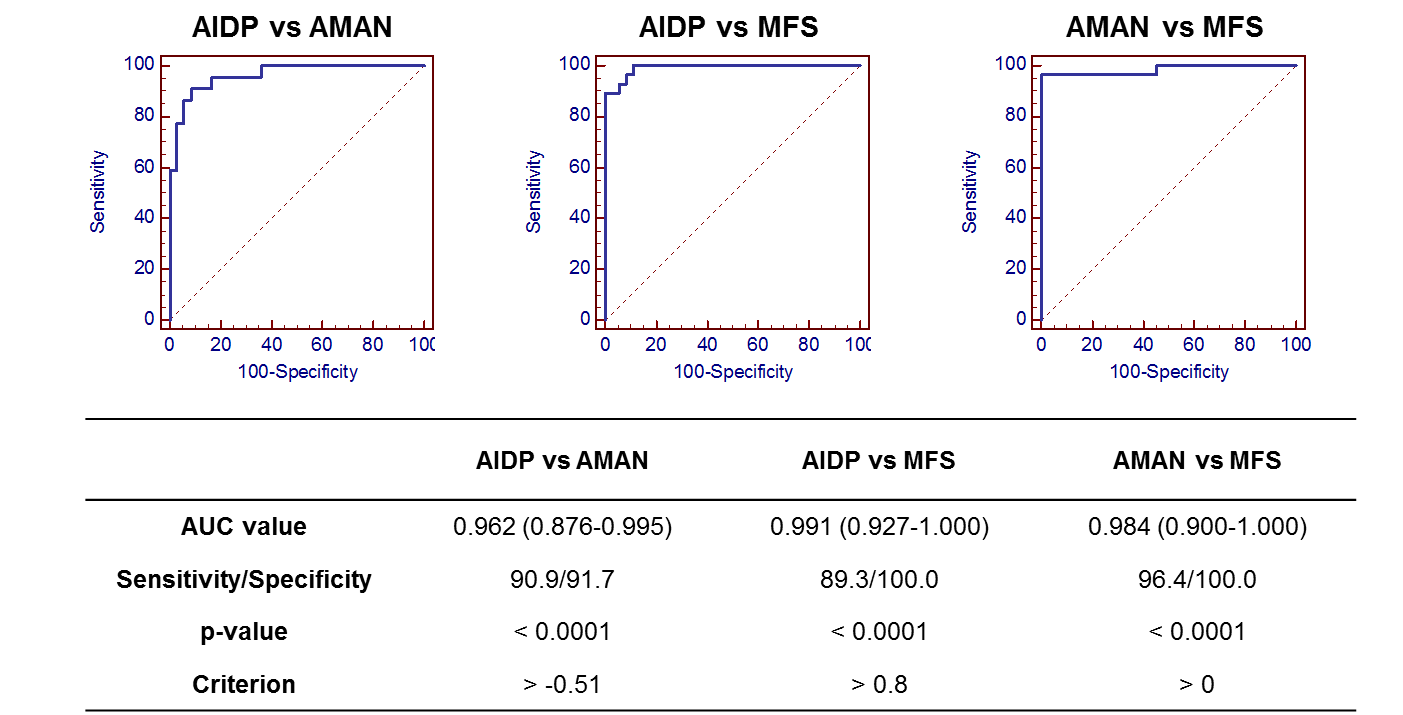


**Fig S5. Receiver operating characteristic (ROC) curve analysis of linearly-formulated biomarker panel by binary logistic regression. Biomarker components are identical to PLS-DA model**


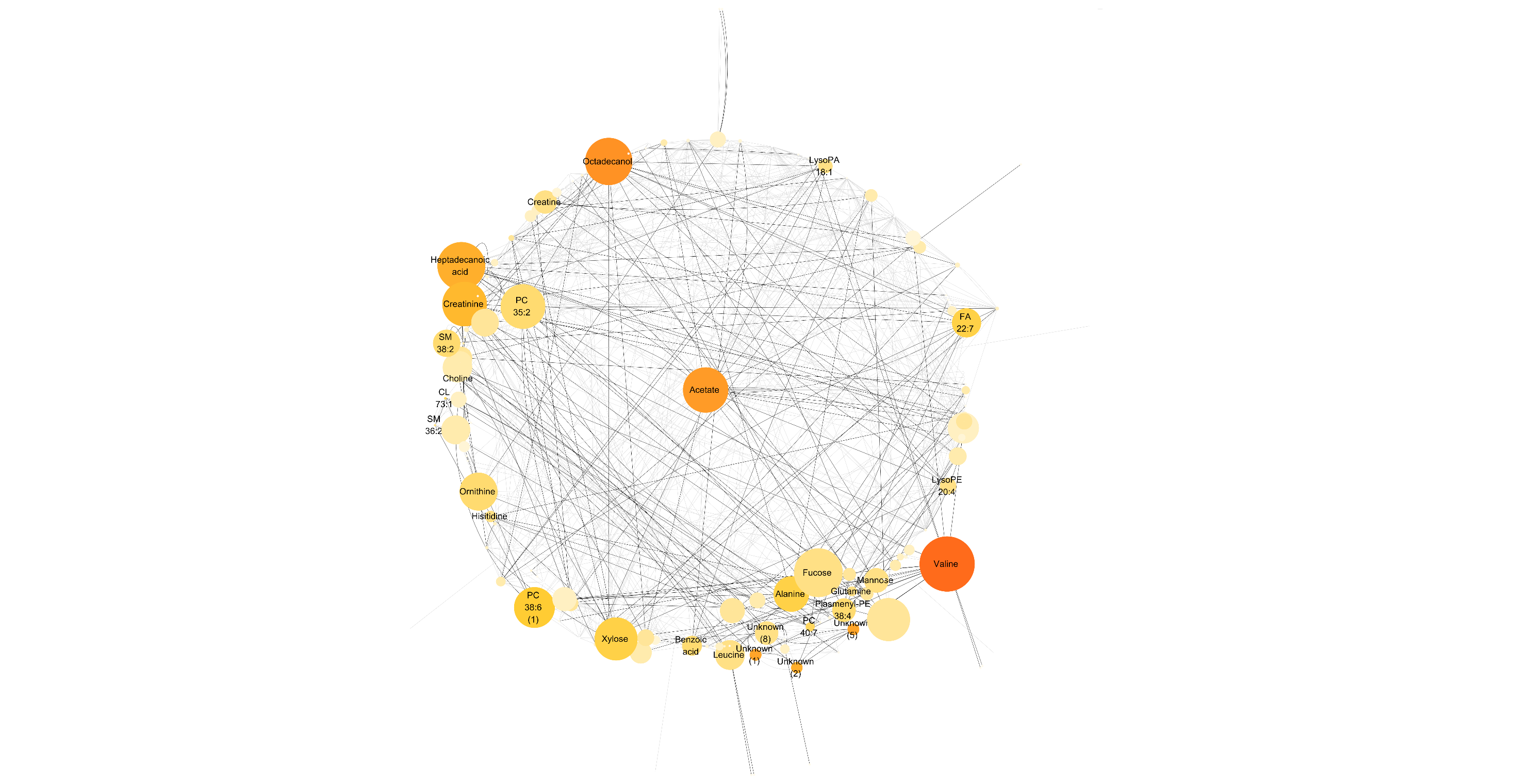

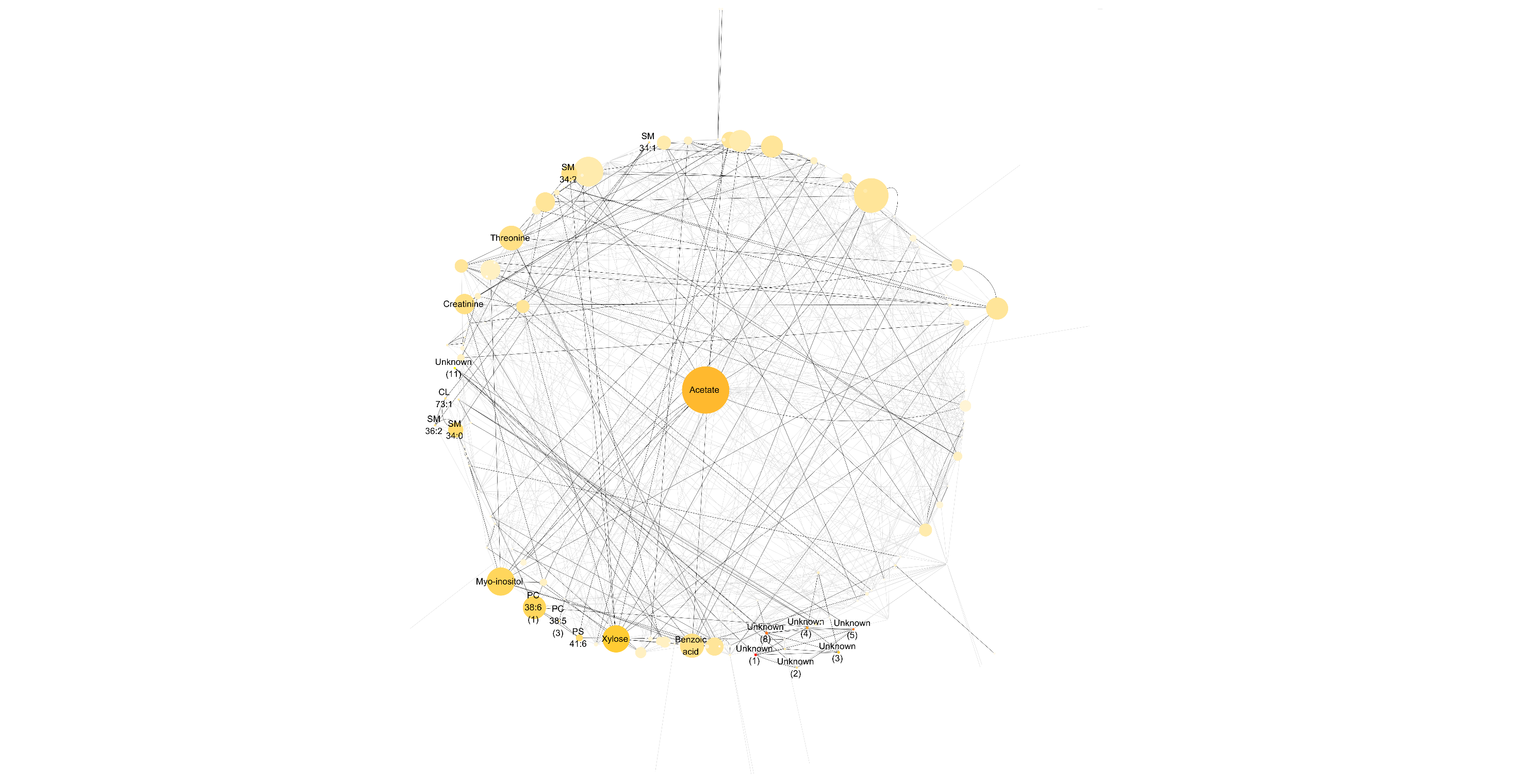

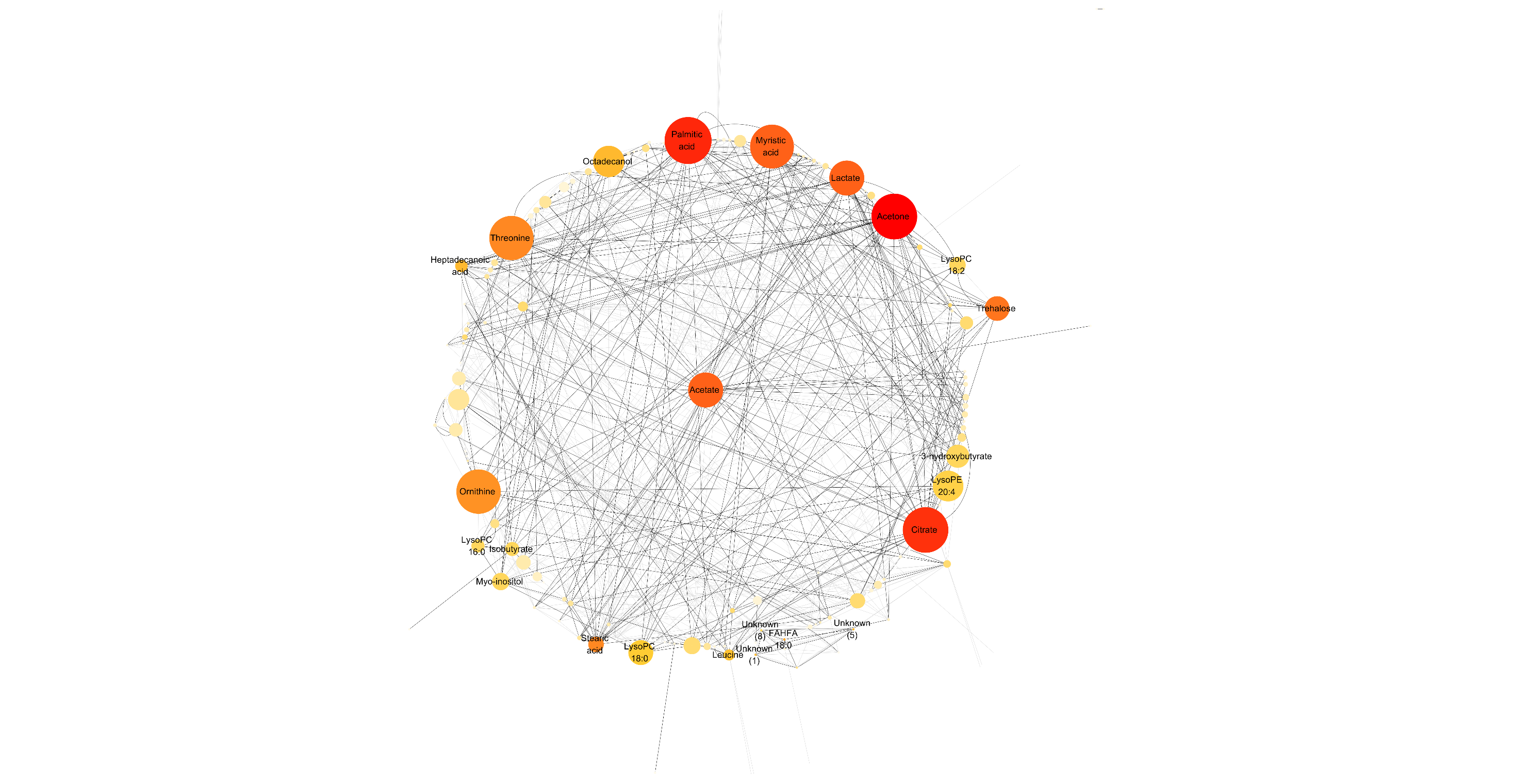

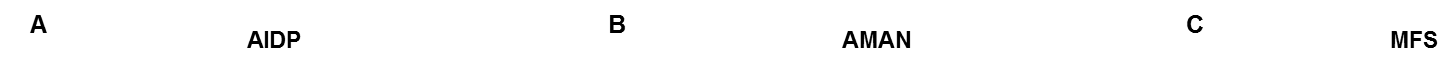


**
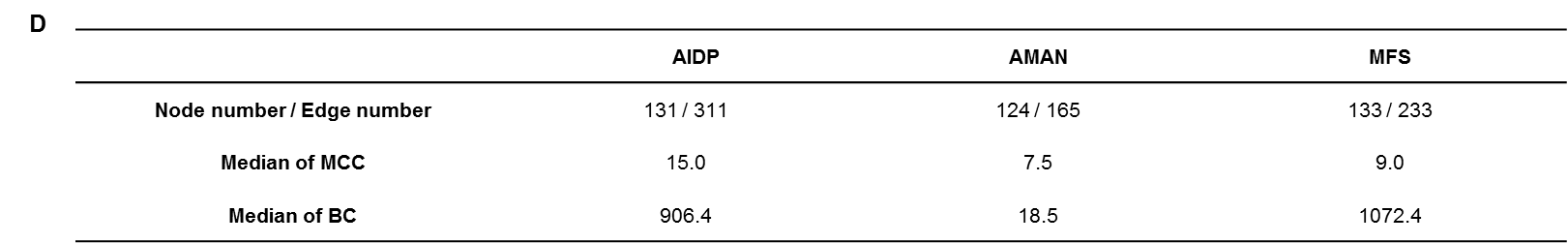
**
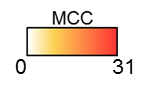


**Fig S6. Integrative analysis of linear correlation network of primary metabolite and lipid molecule (A-C).** Node presented metabolites that are connected by linear relation (edge) estimated by the Bayesian law. The subnetworks consisted of top 20-ranked nodes with maximal clique centrality and extend to their neighbors. Only those of the metabolites were presented with node color and name for visual clarity. Node size is proportional to betweenness centrality. Identical structure (backbone) was applied to all networks for better comparison and thicker edge was used for linear relation for each network. Network topological characteristics are presented in table (D).


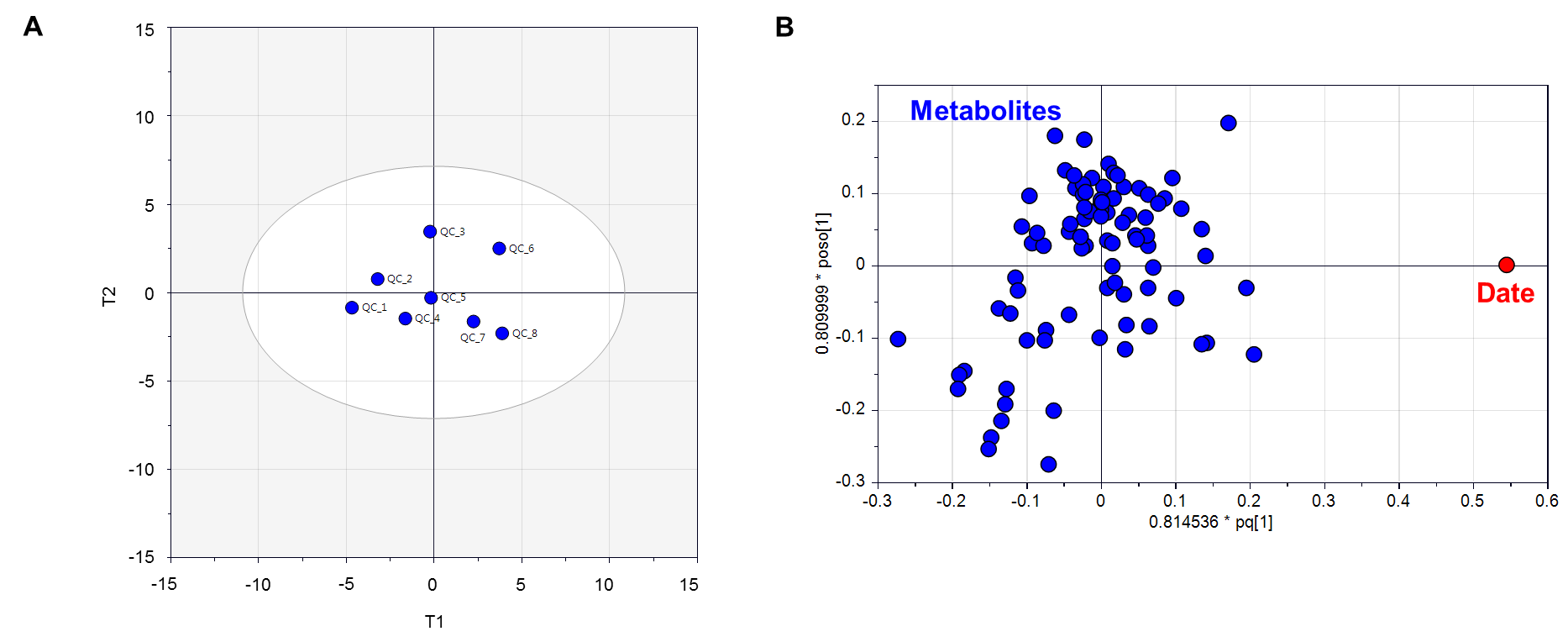


**Fig S7. Quality-check and validation of mass-spectrometric data. (A) Principal component analysis of QC mixture compounds.** Score scatter plot of compound profiles of 8 QC samples with X axis (T1) and Y axis (T2). X and Y-axis indicated the most and the second most discriminant vectors, respectively. The confidence ellipse is based on Hotelling T2, at significance level of 0.05. **(B) Loading scatter plot for potential confounding effect of sample storage period based on orthogonal projection in latent structure (OPLS) analysis.** X variable was set to metabolites whereas y variable was set to the sampling year. The result suggested no significant association between sample storage period and metabolites levels (pq < 0.3)


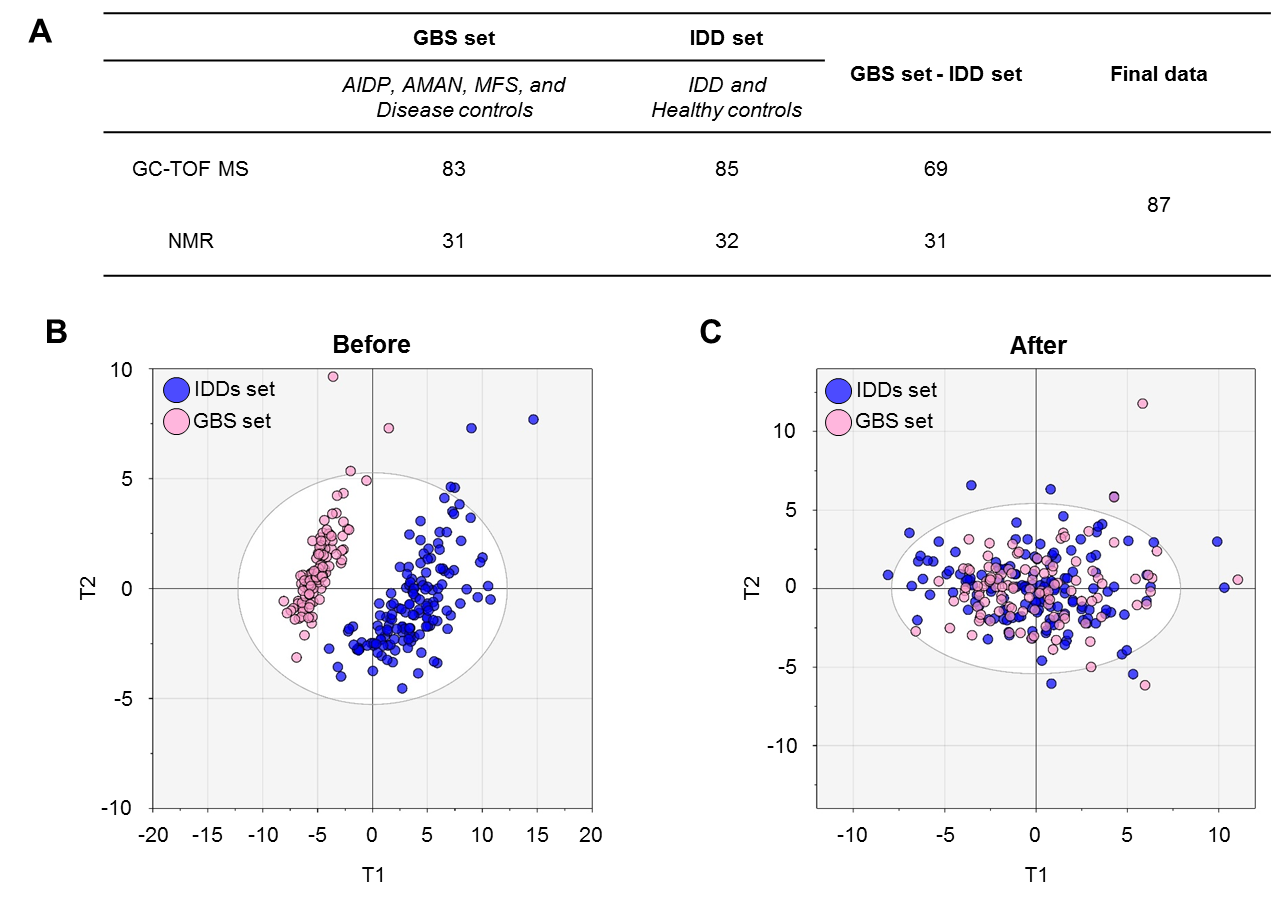


**Fig S8. The number of metabolites according study and analytical platform (A) and batch effect removal using surrogated variable analysis (SVA).** The process was evaluated by score scatter plots using unsupervised multivariate statistical model (PCA) prior to batch effect removal (B) and after batch effect removal (C).


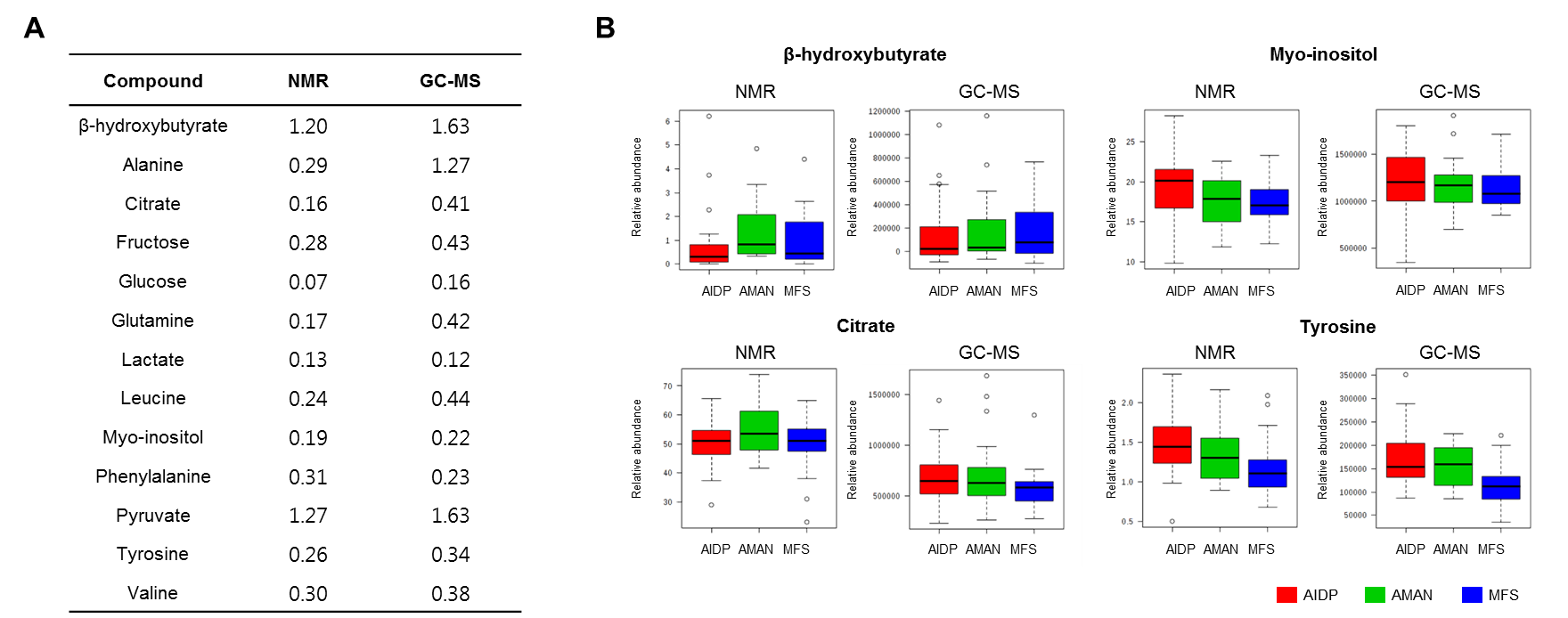


**Fig S9. Comparison of the metabolites that were detected by both GC-TOF MS and NMR spectroscopy** presented by (A) percentage of coefficient variation (%CV) and (B) box-and-whisker plots. %CV was calculated based on the median of %CVs in each disease groups across the metabolites. Relative abundances was visualized for some of the metabolites.

**Supplementary table**

**Supplementary table S1. Statistical significant analysis of GBS-specific metabolites by Student t-test and Kruskal-Wallis test with Benjamini-Hochberg adjustment.**

|  | **GBS** | | | | | **IDD** | | | | | **DC** | | | | |
| --- | --- | --- | --- | --- | --- | --- | --- | --- | --- | --- | --- | --- | --- | --- | --- |
|  | **Compound** | **T-TEST (HC vs GBS)** | | **Kruskal-Wallis** | | **Compound** | **T-TEST (HC vs IDD)** | | **Kruskal-Wallis** | | **Compound** | **T-TEST (HC vs DC)** | | **Kruskal-Wallis** | |
|  |  | **p-value** | **Fold change** | **p-value** | **Adjusted  p-value (Benjamini/ Hochberg)** |  | **p-value** | **Fold change** | **p-value** | **Adjusted  p-value (Benjamini/ Hochberg)** |  | **p-value** | **Fold change** | **p-value** | **Adjusted  p-value (Benjamini/ Hochberg)** |
| **Unique features** | 3-hydroxyisovalerate | 7E-03 | 0.81 | 1E-04 | 4E-04 |  |  |  |  |  |  |  |  |  |  |
|  | Acetate | 2E-09 | 0.72 | 2E-16 | 2E-15 | 2-hydroxybutyrate | 2E-03 | 1.45 | 1E-16 | 1E-15 |  |  |  |  |  |
|  | Acetoacetate | 2E-03 | 2.31 | 1E-11 | 6E-11 | 3-hydroxypropionic acid | 3E-03 | 0.79 | 8E-03 | 2E-02 | 2-hydroxybutyrate | 4E-04 | 0.59 | 1E-16 | 1E-15 |
|  | Acetone | 3E-02 | 0.82 | 0E+00 | 0E+00 | Acetone | 2E-04 | 1.81 | 0E+00 | 0E+00 | Cellobiose | 9E-18 | -0.15 | 0E+00 | 0E+00 |
|  | Choline | 5E-02 | 0.80 | 9E-10 | 4E-09 | Butane-2,3-diol | 4E-03 | 0.18 | 4E-01 | 6E-01 | Heptadecanoic acid | 2E-02 | 0.87 | 5E-04 | 2E-03 |
|  | Fructose | 2E-02 | 0.83 | 6E-03 | 2E-02 | Glucose | 5E-02 | 0.95 | 0E+00 | 0E+00 | Histidine | 3E-03 | 1.41 | 6E-10 | 3E-09 |
|  | Glucose | 1E-03 | 1.08 | 0E+00 | 0E+00 | Isoleucine | 2E-03 | 0.72 | 4E-02 | 8E-02 | Hypoxanthine | 4E-02 | 1.37 | 3E-01 | 5E-01 |
|  | Isobutyrate | 2E-03 | 0.82 | 2E-10 | 1E-09 | Pyroglutamate | 4E-02 | 1.36 | 3E-03 | 9E-03 |  |  |  |  |  |
|  | Leucine | 7E-03 | 0.83 | 2E-09 | 1E-08 |  |  |  |  |  |  |  |  |  |  |
| **Common features** | Creatine | 1E-07 | 0.75 | 0E+00 | 0E+00 |  |  |  |  |  | Trehalose | 8E-17 | -0.15 | 0E+00 | 0E+00 |
|  | Threose | 1E-06 | 0.57 | 1E-10 | 7E-10 |  |  |  |  |  | Creatine | 2E-08 | 0.71 | 0E+00 | 0E+00 |
|  | 1-monostearin | 3E-05 | 2.06 | 2E-06 | 7E-06 |  |  |  |  |  | Lactate | 3E-07 | 0.80 | 0E+00 | 0E+00 |
|  | 3-hydroxyisobutyrate | 6E-05 | 1.48 | 1E-14 | 9E-14 | Threose | 5E-14 | 0.38 | 1E-10 | 7E-10 | Valine | 1E-05 | 0.65 | 2E-13 | 1E-12 |
|  | Lactate | 1E-04 | 0.85 | 0E+00 | 0E+00 | 1-monostearin | 6E-07 | 2.15 | 2E-06 | 7E-06 | 1-monostearin | 3E-05 | 1.82 | 2E-06 | 7E-06 |
|  | Methionine | 2E-04 | 0.53 | 4E-08 | 1E-07 | 1-monopalmitin | 4E-06 | 1.66 | 1E-04 | 4E-04 | Threose | 7E-05 | 0.33 | 1E-10 | 7E-10 |
|  | 1-monopalmitin | 4E-04 | 1.59 | 1E-04 | 4E-04 | Inosine | 2E-03 | 0.72 | 4E-03 | 1E-02 | 3-hydroxyisobutyrate | 7E-05 | 1.47 | 1E-14 | 9E-14 |
|  | Methanol | 1E-03 | 0.68 | 1E-05 | 4E-05 | Methionine | 7E-03 | 0.35 | 4E-08 | 1E-07 | Phenylalanine | 8E-04 | 0.78 | 3E-15 | 3E-14 |
|  | Phenylalanine | 2E-03 | 0.77 | 3E-15 | 3E-14 | Formate | 9E-03 | 1.22 | 4E-02 | 9E-02 | Methanol | 1E-03 | 0.66 | 1E-05 | 4E-05 |
|  | Alanine | 3E-03 | 0.77 | 1E-13 | 7E-13 | Glycolic acid | 3E-02 | 1.52 | 8E-02 | 2E-01 | Inosine | 3E-03 | 0.77 | 4E-03 | 1E-02 |
|  | Valine | 1E-02 | 0.80 | 2E-13 | 1E-12 |  |  |  |  |  | 1-monopalmitin | 3E-03 | 1.56 | 1E-04 | 4E-04 |
|  | Inosine | 2E-02 | 0.80 | 4E-03 | 1E-02 |  |  |  |  |  | Alanine | 3E-03 | 0.79 | 1E-13 | 7E-13 |
|  | Glycolic acid | 4E-02 | 1.47 | 8E-02 | 2E-01 |  |  |  |  |  | Formate | 3E-02 | 1.21 | 4E-02 | 9E-02 |
|  |  |  |  |  |  |  |  |  |  |  | Methionine | 3E-02 | 0.51 | 4E-08 | 1E-07 |
